# Supplementary material for: MERVL/Zscan4 Network Activation Results in Transient Genome-wide DNA Demethylation of mESCs
Source: Cell Rep. 2016 Sep 27;17(1):179–92. doi: 10.1016/j.celrep.2016.08.087 (PMC5055476; doi:10.1016/j.celrep.2016.08.087)

Cell Reports, Volume *17*

Supplemental Information

MERVL/Zscan4 Network Activation Results in Transient Genome-wide DNA Demethylation of mESCs

Mélanie A. Eckersley-Maslin, Valentine Svensson, Christel Krueger, Thomas M. Stubbs, Pascal Giehr, Felix Krueger, Ricardo J. Miragaia, Charalampos Kyriakopoulos, Rebecca V. Berrens, Inês Milagre, Jörn Walter, Sarah A. Teichmann, and Wolf Reik

Supplementary Information

Supplementary Experimental Procedures

Cell Culture medium composition

Serum/LIF: DMEM 4,500 mg/l glucose, 4 mM L-glutamine, 110 mg/l sodium pyruvate, 15% fetal bovine serum, 1 U/ml penicillin, 1 mg/ml streptomycin, 0.1 mM nonessential amino acids, 50 mM b-mercaptoethanol, and 10^3 U/ml LIF.
2i/LIF: 50:50 DMEM/F12:Neurobasal medium (Gibco), 1x N2 supplement (Gibco), 1x B27 supplement (Gibco), L-glutamine, 1 U/ml penicillin, 1 mg/ml streptomycin, 50 mM b-mercaptoethanol, 10^3 U/ml LIF, 1μM PD0325901 inhibitor and 3μM CHIR99021 inhibitor.

Immunofluorescence

Cells were grown on gelatin-coated glass coverslips, fixed in 4% formaldehyde (Polysciences, Inc. Cat #18814) for 20-30 minutes and permeabilised in 0.5% Triton X-100 in PBS for 15-20 minutes at room temperature. Coverslips were blocked in 3% BSA for 1-3 hours at room temperature or overnight at 4 degrees. Primary antibodies were used at the following dilutions: anti-Oct4 (Santa Cruz sc5279) 1:100; anti-Nanog (abcam ab80892) 1:100; anti-Sox2 (abcam ab97959) 1:100; anti-Dnmt1 (abcam ab87654) 1:100; anti-Dnmt3a (Santa Cruz sc20703) 1:50; anti-Dnmt3b (abcam ab13604) 1:500; anti-Dppa4 (Santa Cruz sc74616 1:200). The SUnSET assay was performed as previously described (Schmidt et al., 2009), with a pre-incubation of 1μg/ml puromycin for 10minutes at 37 degrees prior to fixation and detection using anti-puromycin antibody (Millipore MABE343 clone 12D10) at 1:200 dilution. Fluorescently labelled (Alexa488 or Alexa647) secondary antibodies were used at 1:1000 for 30-60 minutes at room temperature, coverslips counterstained with DAPI and mounted in ProLong Gold Antifade mounting medium (Invitrogen P36934). Images were acquired using a Zeiss 780 confocal microscope system with 40x or 63x oil immersion lenses. Image processing was performed using Zeiss ZEN or FUJI software. For semi-quantitative analysis of DNA organisation, nuclei were classified as normal (containing discrete heterochromatic foci) or altered (few large DAPI dense regions), based on DAPI staining. Subsequently, the Zscan4+ or MERVL+ status of the cell was determined.

ATAC-seq analysis

Raw FastQ data were trimmed with Trim Galore to remove Nextera adapters and poor basecall qualities (v0.4.1, default parameters) and mapped to the mouse GRCm38 genome assembly using Bowtie 2 (v2.2.5). Data were quantitated using SeqMonk (www.bioinformatics.babraham.ac.uk/projects/seqmonk/). For promoter analysis, 5kb probes surrounding transcription start site (TSS) were defined and probe trend plots generated. For the bean plots in figure 1F and S1D, probes were generated for each of the features, normalised read counts performed, probe reports generated and RStudio used for plotting (genome: 500bp sliding windows; genic: annotated genes +/- 2kb; inergenic: whole genome – genic regions; promoters: 1kb upstream of TSS; upregulated promoters: promoters of genes differentially expressed as determined by RNA-seq analysis (see below); ERVL, MT2_Mm, MERVL-int, MT2B, MERVL 2A-int, ERVK and LINE L1: appropriate RepeatMasker annotation tracks; CpG islands: SeqMonk annotation track; H3K27ac and H3K4me1 enhancers: peak data from (Creyghton et al., 2010); ESC Super Enhancers: data from (Whyte et al., 2013)). For repeat analysis used in Figure S3D, probes were generated for the filtered repeat track used in RNA sequencing repeat analysis (see below) and normalised read counts (with probe length correction) generated.

RNA sequencing data analysis

Raw FastQ data were trimmed with Trim Galore (v0.4.1, default parameters) and mapped to the mouse GRCm38 genome assembly using TopHat v2.0.12, guided by gene models from Ensembl v70. Data were quantitated at mRNA level using the RNA-seq quantitation pipeline in SeqMonk software (www.bioinformatics.babraham.ac.uk/projects/seqmonk/). Strand specific quantification was performed using mRNA probes and cumulative distributions matched across samples. Differentially expressed genes were determined using DESeq2 (p-value 0.05, with multiple testing correction) and Intensity difference filter (p-value 0.05, with multiple testing correction), with the high-confidence DE genes defined as the intersection between the two statistical tests. The final list of MERVL-driven DE genes was determined by taking the sum of the three DE gene lists (180 genes). Manual filtering removed 8 genes (Fam190a, Tnfrsf22, Gm11052, Cmah, Arsk, Gm16344, Fgf1, Ac165327.2) as their exons overlapped with MERVL elements which did not continue to be successfully spliced to the remainder of the gene, giving a final high confidence list of 172 MERVL-driven differentially expressed genes used in all downstream analyses.
Repeat annotations for the mouse GRCm38 genome build (generated by RepeatMasker) were downloaded from the UCSC website (Oct 2015). The genomic sequences of all instances of different repeat families (LTR ERV1, LTR ERVL, LINE_L1, LINE_L2 and SINE_B2 etc.) were extracted and concatenated into repeat family

pseudo-genomes, whereby individual repeat instances were padded by 'NNNNN' to prevent reads from aligning over artificially created repeat boundaries. Read 1 files of RNA-Seq datasets were then aligned against the repeat genomes using Bowtie (v1.0.1; default parameters) and alignments to repeat families were scored. Graphing and statistics was performed using Excel or RStudio.

Repeat analysis
The analysis of repetitive genomic regions in our data was performed by a dual approach: A) RNA-Seq reads were mapped against the mouse genome build GRCm38 using Tophat (v2.1.0 with Bowtie 2, v2.2.5) specifying that if a read mapped more than once, it would be assigned one of the genomic locations. Repetitive sequences were then analysed using RepeatMasker annotation. For the global analysis, repeat masker annotations were filtered to be at least 100bp long, and to have at least 10 reads either in our own data, or in RNA-seq data published in (Akiyama et al., 2015). For the analysis of individual repeat classes, no filtering was applied. B) We also wanted to include sequences that are not present in the mouse genome assembly, namely major and minor satellites and telomeres. For major and minor satellites we used the sequences specified in (Akiyama et al., 2015). For LINE, SINE, ERV1, MaLR, ERVK and ERVL elements, genomic sequences specified by RepeatMasker (http://www.repeatmasker.org) were downloaded from the UCSC website and concatenated into repeat class pseudo-genomes with individual repeat instances padded by 'NNNNN' to prevent reads from aligning over artificially created boundaries. Similarly, the telomeric hexamer repeat was concatenated to a total length of 300 bp. Alignments were performed using Bowtie2 (v2.2.5). Data shown in Figure 1G was created using approach A, data shown in Figure 1F was created using approach B.

Single-cell RNA sequencing sorting strategy
We sorted four 96-well plates where each plate had five populations: 56 cells in a MERVL+Zscan4c+ population, 16 cells in a Zscan4c+ only population, 8 cells in a negative population with slight MERVL expression, 14 cells from a completely negative populations, and 2 empty wells for control. To attempt to combat potential batch effects during the library preparation, wells were transferred to new plates mixing rows from different sorting plates in to same library plate.

Single cell RNA-sequencing library preparation by SMART-seq v2
Single-cells were sorted in 2uL of Lysis Buffer (1:20 solution of RNase Inhibitor (Clontech, cat. no. 2313A or Invitrogen RNAse OUT) in 0.2% v/v Triton X-100 (Sigma-Aldrich, cat. no. T9284) in 96 well plates, spun down and immediately frozen at -80 degrees. Oligo-dT primer, dNTPs (ThermoFisher, cat. no. 10319879) and ERCC RNA Spike-In Mix (1:25,000,000 final dilution, Ambion, cat. no. 4456740) were then added, and Reverse Transcription and PCR were performed as in (Picelli et al., 2014). The cDNA libraries for sequencing were prepared using Nextera XT DNA Sample Preparation Kit (Illumina, cat. no. FC-131-1096), according to the protocol supplied by Fluidigm (PN 100-5950 B1). Libraries from 96 single cells were pooled and purified using AMPure XP beads (Beckman Coulter). Pooled samples were sequenced on an Illumina HiSeq 2500 instrument, using paired-end 100-bp reads.

Single cell RNA-sequencing data analysis and quality control
The gene expressions for every sample were quantified using Salmon version 0.5.0 (Patro et al., 2015) with library type parameter IU, with a transcriptome index built from the Ensembl 78 cDNA annotation (GRCm38 patch 3), together with ERCC transcript sequences. The index also contained 313 sequences for Mus musculus specific repeats from RepBase (Jurka et al., 2005) for assessing potentially transcribed repeats (such as MERVL). By comparing largely technical features with the 8 empty, control wells, in the plates we filtered out 65 of the 376 samples based on ERCC spike in ratio (more than 60%) and number of sequenced read pairs (less than 100,000). The thresholds were consistent with other technical features such as mapping rate, mitochondrial content and number of detected genes. This left 319 samples that we considered as healthy single cells, and used for further analysis.
From the Transcripts per million (TPM) of any sample, we removed the spike-in expression and rescaled the values of the endogenous genes to sum to a million. This *endogenous TPM* represents the relative abundance of a gene within a cell. For differential expression testing we used linear modelling and the likelihood ratio test for significance analysis, where we controlled for the number of observed genes in a sample.

Pseudotime ordering of scRNA-seq data
To investigate the dynamics of the MERVL network, we created a trajectory (or "pseudotime") over the expression of Zscan4 (summed TPM for Zscan4b-f) and MERVL for the two positive conditions, using a Bayesian Gaussian Process Latent Variable Model (Titsias and Lawrence, 2010). Given the transcriptome data, it is not possible to distinguish individual genomic copies of MERVL and as such the total MERVL expression was analysed. To identify genes with dynamic expression over this trajectory, we fitted two Gaussian Processes

for every gene; one with a Bias kernel (which assumes all expression changes are due to noise), and one with a Squared Exponential + Bias kernel (which can also handle dynamic changes in addition to noise). We ranked genes based on the ratio of the optimized likelihoods for the models. We considered 297 genes significantly dynamic based on this measure. These dynamic genes were then clustered using the Mixtures of Hierarchical Gaussian Processes model to groups of genes with a similar common expression pattern over the trajectory (Hensman et al., 2015).

Cell cycle analysis

To assess what states of cell cycle where represented in the different conditions of scRNA-Seq data we used the Pairs method in the Cyclone tool (Scialdone et al., 2015). We ran the method on the TPM values of the data.

Bisulfite sequencing analysis

Raw sequence reads were trimmed to remove both poor quality calls and adapters using Trim Galore (v0.4.1, www.bioinformatics.babraham.ac.uk/projects/trim_galore/, Cutadapt version 1.8.1, parameters: --paired). Trimmed reads were first aligned to the mouse genome in paired-end mode to be able to use overlapping parts of the reads only once while writing out unmapped singleton reads; in a second step remaining singleton reads were aligned in single-end mode. Alignments were carried out with Bismark v0.14.4 (Krueger and Andrews, 2011) with the following set of parameters: a) paired-end mode: --pbat; b) single-end mode for Read 1: --pbat; c) single-end mode for Read 2: defaults.

Reads were then deduplicated with deduplicate_bismark selecting a random alignment for position that were covered more than once. CpG methylation calls were extracted from the deduplicated mapping output ignoring the first 6bp of each read to reduce the methylation bias typically observed in PBAT libraries using the Bismark methylation extractor (v0.14.4) with the following parameters: a) paired-end mode: --ignore 6 --ignore_r2 6; b) single-end mode: --ignore 6.

Data were quantitated using SeqMonk (www.bioinformatics.babraham.ac.uk/projects/seqmonk/). Probes were defined to contain 50 CpGs each with a minimum read count of 4 and percentage methylation determined on the pooled replicate data. For analysis of specific genome features these were defined as follows: Gene bodies (probes overlapping genes), Promoters (probes overlapping 1000bp upstream of genes), CGI promoters (promoters containing or within 250bp of a CGI), non-CGI promoters (all other promoters), LMRs (Stadler et al., 2011), H3K27ac and H3K4me1 Enhancers (Creyghton et al., 2010), Super-enhancers (Whyte et al., 2013). For repetitive elements, Bismark (v0.14.4, using Bowtie 2, default parameters) was used to map all reads from each data set against consensus sequences constructed from Repbase (Jurka et al., 2005). The methylation level was expressed as the mean of individual CG sites. Graphing and statistics was performed using Excel or RStudio.

Methylation quantification by PBAT

For global methylation level quantification, whole genome bisulfite libraries were generated using a post-bisulfite adapter tagging (PBAT) method using 10 cycles of amplification. Libraries were sequenced at low coverage generating ~20-30x10^5 aligned reads per sample. Raw sequence reads were processed as above. 50kb probes (minimum 1 read, minimum 1 observation) were defined and mean methylation level determined for each sample.

Mass Spectrometry

Genomic DNA quantified using picogreen assay (Invitrogen) was digested using DNA Degradase plus (Zymo Research) for 90 minutes at 37 degrees and analysed by liquid chromatography-tandem mass spectrometry on a LTQ Orbitrap Velos mass spectrometer (Thermo Scientific) fitted with a nanoelectrospray ion-source (Proxeon, Odense, Denmark). Mass spectral data for cytosine, 5-methylcytosine and 5-hydroxymethylcytosine were acquired as previously described (Ficz et al., 2013).

Hairpin Bisulfite sequencing

Hairpin bisulfite sequencing allows the analysis of both DNA strands of one individual chromosome giving detailed information about the methylation patterns of cells. Genomic DNA is cut using 10U of the restriction enzyme Eco471 for major satellites (mSat; GSAT_MM) and murine endogenous retrovirus-like (MERVL) and 10U of BsaWI for a class of Long Interspersed Nuclear Elements (L1mdT). After heat inactivation of the restriction enzymes 200U T4 DNA Ligase, 10mM ATP and 100µM hairpin oligo nucleotide are added directly to the reaction and incubated for overnight at 16°C. The ligation covalently links both DNA strands with each other. In the following step the reaction is subjected to a bisulfite treatment which was performed using the EZ Methylation-GoldTM Kit from Zymo Research. The treated DNA was amplified by PCR to create amplicons for each repetitive element. Sequencing was performed on a MiSeq Illumina platform with 2x300bp. The Analysis was carried out with the BiQAnalyzer HT and python script. To calculate the efficiencies of de novo

and maintenance methylation we applied an extension of the Hidden Markov Model described in (Arand et al., 2012), allowing de-novo methylation to happen in both hemi and unmethylated CpG sites with the same probability. Primer sequences used are as follows:

MERVL-HP
(CGCCCGAGACAAGGTGATTCTAGTTATTATAATGGACAGCGTAGACAAAAGAATGTTTATAATAA
CATACCCAGTAATGGTCAGCACAGGAGAGGTGAAATTTATAATGGCATGACTCGGTTGgwtgggRttat
ddddddddatgggRttgwTTCAACCGAGTCATGCCATTATAAATTTCACCTCTCCTGTGCTGACCATTACTG
GGTATGTTATTATAAACATTCTTTTGTCTACGCTGTCCATTATAATAACTAGAATCACCTTGTCTCG
GGCG);
L1HP
(CCCGGGACCAAGATGGCGACCGCTGCTGCTGTGGCTTAGGCCGCCTCCCCAGCCGGGTGGGCACC
TGTCCTttGGaGGGRttATNNNNNNNNATGGGRtttCCGGAGGACAGGTGCCCACCCGGCTGGGGAGGC
GGCCTAAGCCACAGCAGCAGCGGTCGCCATCTTGGTCCCGGG);
mSat-HP
(GgaaaatttagaaatgtttaatgtaggaCGtggaatatggcaagaaaactgaaaatcatgggaaatgagaaacatccacttgtCGacttgaaaaatgaCGaa
atcactaaaaaaCGtgaaaaatgagaaatgcacactgaaggNTgggRTTatNNNNNNNNatgggRTTgNccttcagtgtgcatttctcatttttca
CGttttttagtgatttCGtcatttttcaagtCGacaagtggatgtttctcatttttatgatttttagtttttttgtt).

Statistical Methods

Statistics were performed using Seqmonk (www.bioinformatics.babraham.ac.uk/projects/seqmonk/), Excel, Graphpad prism or RStudio. For mass spectrometry, a paired t-test was used to determine p-value between measurements made from matched MERVL+Zscan4+ and negative sorted cells on different days. For CpG methylation levels determined by PBAT, a homoscedastic two-tailed t-test was used to determine significance between at least 3 biological replicates for each sample. For statistical analysis of transcription of individual genes, expression levels were quantified in the RNA-sequencing data as above and a homoscedastic two-tailed t-test performed between at least 3 biological replicates per sample. Semi-quantitative immunofluorescence measurements were performed using ImageJ in a blind manner and Chi-square test performed in Excel to determine significance.

Supplemental References

Akiyama, T., Xin, L., Oda, M., Sharov, A.A., Amano, M., Piao, Y., Cadet, J.S., Dudekula, D.B., Qian, Y., Wang, W., Ko, S.B.H., Ko, M.S.H., 2015. Transient bursts of Zscan4 expression are accompanied by the rapid derepression of heterochromatin in mouse embryonic stem cells. DNA Res. dsv013. doi:10.1093/dnares/dsv013

Arand, J., Spieler, D., Karius, T., Branco, M.R., Meilinger, D., Meissner, A., Jenuwein, T., Xu, G., Leonhardt, H., Wolf, V., Walter, J., 2012. In vivo control of CpG and non-CpG DNA methylation by DNA methyltransferases. PLoS Genet. 8, e1002750. doi:10.1371/journal.pgen.1002750

Buettner, F., Natarajan, K.N., Casale, F.P., Proserpio, V., Scialdone, A., Theis, F.J., Teichmann, S.A., Marioni, J.C., Stegle, O., 2015. Computational analysis of cell-to-cell heterogeneity in single-cell RNA-sequencing data reveals hidden subpopulations of cells. Nat. Biotechnol. 33, 155–160. doi:10.1038/nbt.3102

Creyghton, M.P., Cheng, A.W., Welstead, G.G., Kooistra, T., Carey, B.W., Steine, E.J., Hanna, J., Lodato, M.A., Frampton, G.M., Sharp, P.A., Boyer, L.A., Young, R.A., Jaenisch, R., 2010. Histone H3K27ac separates active from poised enhancers and predicts developmental state. Proc. Natl. Acad. Sci. U.S.A. 107, 21931–21936. doi:10.1073/pnas.1016071107

Deng, Q., Ramsköld, D., Reinius, B., Sandberg, R., 2014. Single-cell RNA-seq reveals dynamic, random monoallelic gene expression in mammalian cells. Science 343, 193–196. doi:10.1126/science.1245316

Domcke, S., Bardet, A.F., Adrian Ginno, P., Hartl, D., Burger, L., Schübeler, D., 2015. Competition between DNA methylation and transcription factors determines binding of NRF1. Nature 528, 575–579. doi:10.1038/nature16462

Ficz, G., Hore, T.A., Santos, F., Lee, H.J., Dean, W., Arand, J., Krueger, F., Oxley, D., Paul, Y.-L., Walter, J., Cook, S.J., Andrews, S., Branco, M.R., Reik, W., 2013. FGF signaling inhibition in ESCs drives rapid genome-wide demethylation to the epigenetic ground state of pluripotency. Cell Stem Cell 13, 351–359. doi:10.1016/j.stem.2013.06.004

Hensman, J., Rattray, M., Lawrence, N.D., 2015. Fast Nonparametric Clustering of Structured Time-Series. IEEE Trans Pattern Anal Mach Intell 37, 383–393. doi:10.1109/TPAMI.2014.2318711

Jurka, J., Kapitonov, V.V., Pavlicek, A., Klonowski, P., Kohany, O., Walichiewicz, J., 2005. Repbase Update, a database of eukaryotic repetitive elements. Cytogenet. Genome Res. 110, 462–467. doi:10.1159/000084979

Kolodziejczyk, A.A., Kim, J.K., Tsang, J.C.H., Ilicic, T., Henriksson, J., Natarajan, K.N., Tuck, A.C., Gao, X., Bühler, M., Liu, P., Marioni, J.C., Teichmann, S.A., 2015. Single Cell RNA-Sequencing of Pluripotent States Unlocks Modular Transcriptional Variation. Cell Stem Cell 17, 471–485. doi:10.1016/j.stem.2015.09.011

Krueger, F., Andrews, S.R., 2011. Bismark: a flexible aligner and methylation caller for Bisulfite-Seq applications. Bioinformatics 27, 1571–1572. doi:10.1093/bioinformatics/btr167

Kumar, R.M., Cahan, P., Shalek, A.K., Satija, R., DaleyKeyser, A.J., Li, H., Zhang, J., Pardee, K., Gennert, D., Trombetta, J.J., Ferrante, T.C., Regev, A., Daley, G.Q., Collins, J.J., 2014. Deconstructing transcriptional heterogeneity in pluripotent stem cells. Nature 516, 56–61. doi:10.1038/nature13920

Patro, R., Duggal, G., Kingsford, C., 2015. Accurate, fast, and model-aware transcript expression quantification with Salmon, bioRxiv. doi:10.1101/021592

Picelli, S., Faridani, O.R., Björklund, A.K., Winberg, G., Sagasser, S., Sandberg, R., 2014. Full-length RNA-seq from single cells using Smart-seq2. Nat Protoc 9, 171–181. doi:10.1038/nprot.2014.006

Schmidt, E.K., Clavarino, G., Ceppi, M., Pierre, P., 2009. SUnSET, a nonradioactive method to monitor protein synthesis. Nat Meth 6, 275–277. doi:10.1038/nmeth.1314

Scialdone, A., Natarajan, K.N., Saraiva, L.R., Proserpio, V., Teichmann, S.A., Stegle, O., Marioni, J.C., Buettner, F., 2015. Computational assignment of cell-cycle stage from single-cell transcriptome data. Methods 85, 54–61. doi:10.1016/j.ymeth.2015.06.021

Stadler, M.B., Murr, R., Burger, L., Ivanek, R., Lienert, F., Schöler, A., van Nimwegen, E., Wirbelauer, C., Oakeley, E.J., Gaidatzis, D., Tiwari, V.K., Schübeler, D., 2011. DNA-binding factors shape the mouse methylome at distal regulatory regions. Nature 480, 490–495. doi:10.1038/nature10716

Titsias, M.K., Lawrence, N.D., 2010. Bayesian Gaussian Process Latent Variable Model. Proceedings of the th International Conference on Artificial Intelligence and Statistics AISTATS 1–8.

Wang, L., Zhang, J., Duan, J., Gao, X., Zhu, W., Lu, X., Yang, L., Zhang, J., Li, G., Ci, W., Li, W., Zhou, Q., Aluru, N., Tang, F., He, C., Huang, X., Liu, J., 2014. Programming and inheritance of parental DNA methylomes in mammals. Cell 157, 979–991. doi:10.1016/j.cell.2014.04.017

Whyte, W.A., Orlando, D.A., Hnisz, D., Abraham, B.J., Lin, C.Y., Kagey, M.H., Rahl, P.B., Lee, T.I., Young, R.A., 2013. Master transcription factors and mediator establish super-enhancers at key cell identity genes. Cell 153, 307–319. doi:10.1016/j.cell.2013.03.035

Xue, Z., Huang, K., Cai, C., Cai, L., Jiang, C.-Y., Feng, Y., Liu, Z., Zeng, Q., Cheng, L., Sun, Y.E., Liu, J.-Y.,

Horvath, S., Fan, G., 2013. Genetic programs in human and mouse early embryos revealed by single-cell RNA sequencing. Nature 500, 593–597. doi:10.1038/nature12364

Yue, F., Cheng, Y., Breschi, A., Vierstra, J., Wu, W., Ryba, T., Sandstrom, R., Ma, Z., Davis, C., Pope, B.D., Shen, Y., Pervouchine, D.D., Djebali, S., Thurman, R.E., Kaul, R., Rynes, E., Kirilusha, A., Marinov, G.K., Williams, B.A., Trout, D., Amrhein, H., Fisher-Aylor, K., Antoshechkin, I., DeSalvo, G., See, L.-H., Fastuca, M., Drenkow, J., Zaleski, C., Dobin, A., Prieto, P., Lagarde, J., Bussotti, G., Tanzer, A., Denas, O., Li, K., Bender, M.A., Zhang, M., Byron, R., Groudine, M.T., McCleary, D., Pham, L., Ye, Z., Kuan, S., Edsall, L., Wu, Y.-C., Rasmussen, M.D., Bansal, M.S., Kellis, M., Keller, C.A., Morrissey, C.S., Mishra, T., Jain, D., Dogan, N., Harris, R.S., Cayting, P., Kawli, T., Boyle, A.P., Euskirchen, G., Kundaje, A., Lin, S., Lin, Y., Jansen, C., Malladi, V.S., Cline, M.S., Erickson, D.T., Kirkup, V.M., Learned, K., Sloan, C.A., Rosenbloom, K.R., Lacerda de Sousa, B., Beal, K., Pignatelli, M., Flicek, P., Lian, J., Kahveci, T., Lee, D., Kent, W.J., Ramalho-Santos, M., Herrero, J., Notredame, C., Johnson, A., Vong, S., Lee, K., Bates, D., Neri, F., Diegel, M., Canfield, T., Sabo, P.J., Wilken, M.S., Reh, T.A., Giste, E., Shafer, A., Kutyavin, T., Haugen, E., Dunn, D., Reynolds, A.P., Neph, S., Humbert, R., Hansen, R.S., De Bruijn, M., Selleri, L., Rudensky, A., Josefowicz, S., Samstein, R., Eichler, E.E., Orkin, S.H., Levasseur, D., Papayannopoulou, T., Chang, K.-H., Skoultchi, A., Gosh, S., Disteche, C., Treuting, P., Wang, Y., Weiss, M.J., Blobel, G.A., Cao, X., Zhong, S., Wang, T., Good, P.J., Lowdon, R.F., Adams, L.B., Zhou, X.-Q., Pazin, M.J., Feingold, E.A., Wold, B., Taylor, J., Mortazavi, A., Weissman, S.M., Stamatoyannopoulos, J.A., Snyder, M.P., Guigó, R., Gingeras, T.R., Gilbert, D.M., Hardison, R.C., Beer, M.A., Ren, B., Mouse ENCODE Consortium, 2014. A comparative encyclopedia of DNA elements in the mouse genome. Nature 515, 355–364. doi:10.1038/nature13992

Supplemental Figure Legends

Supplemental Figure 1 (related to Figure 1)
(A) Scatterplot showing normalised, $\log_2$ transformed read counts mapping to specific genomic locations of repetitive sequence. Individual repeat classes are highlighted: MERVL (MT2_Mm + MERVL-int, red), LINE L1 (blue) and IAPEZ (IAPEZ-int, black).
(B) Semi-quantitative analysis of DNA organisation. Nuclei were classified according to their DAPI signal as normal (light grey) or altered (dark grey), and expression of the Zscan4 and MERVL reporters. Differences are statistically significant (chi-squared test: Zscan4 χ^2 6.34 x 10^{-113}; MERVL χ^2 5.22 x 10^{-30}).
(C) Chromatin accessibility of different genomic features as determined by ATAC-seq analysis in negative sorted (grey) and MERVL+Zscan4c+ (dark blue) cells. Bars represent mean levels of accessibility.
(D) Log_{10} TPM (transcripts per million) values of MERVL (x-axis) and Zscan4 cluster (y-axis) of single cells from published unsorted mES single cell datasets (Buettner et al., 2015; Kolodziejczyk et al., 2015; Kumar et al., 2014). Only the 204 cells expressing the Zscan4 cluster are shown.
(E) Smoothened heatmap showing expression levels of 172 differentially expressed genes identified by total RNA-sequencing. Each column represents a single-cell (total n=204) that expresses the Zscan4 cluster from published unsorted mES single cell datasets (Buettner et al., 2015; Kolodziejczyk et al., 2015; Kumar et al., 2014). Cells are ordered based on MERVL expression. Median Spearman rank correlation was 0.3 between MERVL and differentially expressed genes, and 0.0 between MERVL and all genes.
(F) Venn diagram showing overlap between differentially expressed genes identified by total RNA-sequencing and dynamic genes over pseudotime (see supplemental experimental procedures for details).

Supplemental Figure 2 (related to Figure 2)
(A-C) Heat map showing relative expression levels of MERVL-LTR driven transcriptional network in early embryos (A,B) and somatic tissues (C). BAT = brown adipose tissue, MEF = mouse embryonic fibroblasts (MEF). Scale bar depicts log relative expression from low (blue) to high (red). Data from (Deng et al., 2014) (A), (Xue et al., 2013) (B) and (Yue et al., 2014) (C).

Supplemental Figure 3 (related to Figure 3)
(A) Consistency of methylation pattern within individual reads containing at least 3 CpGs. Reads were classified as completely methylated (dark grey, p-value 2.3 x10^{-5}), mixed methylation (medium grey, p-value 2.1x10^{-4}) or completely unmethylated (light grey, p-value 6.8x10^{-4}). Error bars represent standard deviation of three biological replicates. *** all three categories of comparisons between negative and MERVL+Zscan4c+ cells are statistically significant (homoscedastic two-tailed t-test).
(B) Bean plots showing distribution of methylation levels for different genome features between negative sorted (grey) and MERVL+Zscan4c+ (blue) cells. Lines represent mean values.
(C) Methylation levels across the Igf2r imprint DMR (chr17:12731191-12752684). Top track shows gene structure of Igf2r (not all gene is shown). Methylation levels for oocyte (red) and sperm (light blue) allow identification of DMR (highlighted in yellow). Oocyte and sperm data from (Wang et al., 2014). Negative ESC (grey) and MERVL+Zscan4c+ ESC (M+Z+, dark blue) tracks show methylation levels across the region (20 CpGs with min coverage of 4 per probe). Bottom track shows overlay between Negative and MERVL+Zscan4c+ datasets.

Supplemental Figure 4 (related to Figure 4)
(A) Flow cytometry analysis showing percentage of MERVL::tdTomato+ and/or Zscan4c::eGFP+ cells in serum and 2i/LIF culture conditions. Differences are statistically significant (2-tailed equal variance t-test, n=4-6).
(B) Quantitative real-time RT-PCR of six MERVL-LTR driven genes in cells cultured in serum or 2i/LIF conditions. Bars represent average of 3 biological replicates +/- standard deviation. Expression levels in serum conditions are set to 1.
(C) Scatterplot showing normalised $\log_2$ transformed read counts for all genes (grey) and MERVL-LTR promoted genes (blue) between wild-type (x-axis) and DNMT TKO (y-axis) ESCs. Data reanalysed from (Domcke et al., 2015).
(D) Flow cytometry plots showing MERVL::tdTomato (y-axis) versus Zscan4c::eGFP (x-axis) reporter expression in steady state conditions (left plot) and of cells sorted from the negative gate (red) and placed back in culture for 6 hours (second plot from left), 24 hours (second plot from right) or 48 hours (right plot). 10,000 cells are shown in each plot. Gates used for subsequent sorting of newly arising MERVL+Zscan4+ cells for methylation analysis are shown in green. The 6 hour time point used a larger gate due to the very small number of newly arising MERVL+Zscan4+ cells.

Supplemental Figure 5 (related to Figure 5)
(A) Immunofluorescence staining of Nanog (top) and Sox2 (bottom) proteins in cells labelled using MERVL::tdTomato reporter (left panel, red). Images represent single confocal images. DNA is stained with DAPI (blue). Scale bar represents 10μm.
(B) Chromosome view of Eif1a-like cluster showing expression levels in negative-sorted (top row) and MERVL+Zscan4c+ (bottom row) cells. Position of genes are denoted (red = sense, blue = antisense), along with opposing strand specific RNA-sequencing reads (sense transcription shows blue, antisense transcription shows red). Bars represent average expression levels of at least 3 biological replicates + standard deviation. Upregulated genes, corresponding to Eif1a-lie genes, are highlighted in red.
(C) Heat map showing expression of genes within cluster on Chromosome 12 (87473449-88356013) between negative-sorted and MERVL+Zscan4c+ cells.

Supplemental Figure 6 (related to Figure 6)
(A) Computation modelling (dashed lines) of hairpin bisulfite data (solid lines) for all biological replicate pairs. Three separate repeat regions were analysed: LINE L1 (top), MERVL (middle) and Major Satellites (bottom) repeats. In each graph, individual reads are classified as fully methylated (orange), hemi-methylated on the upper strand (dark green), hemi-methylated on the lower strand (light green) or unmethylated (blue) reads.

Supplemental Tables

Supplemental Table 1. Expression levels of differentially expressed genes, related to Figure 1.
List of differentially expressed genes between Negative (MERVL-Zscan4-), Zscan4+ only (MERVL-Zscan4+) and MERVL+Zscan4+ sorted cells, along with their chromosome coordinates, ENSEMBL gene ID, Description and average log_2 expression value for the replicates across the three conditions.

Supplemental Table 2. Expression levels of all assessed genes, related to Figure 1.
List of all genes, chromosome coordinates, ENSEMBL gene ID, Description and average log_2 expression value across the Negative (MERVL-Zscan4-), Zscan4+ only (MERVL-Zscan4+) and MERVL+Zscan4+ sorted cells.

Supplemental Table 3. List of genes dynamic over pseudotime, related to Figure 1.
List of transcripts contained within the 5 clusters that are dynamic over pseudotime as determined by single-cell RNA sequencing analysis (see Supplemental Experimental Procedures).

Supplemental Table 4. Expression levels of differentially expressed genes in other datasets, related to Figure 2.
Expression levels for the 172 MERVL-LTR driven genes in the different datasets analysed. (A) Park et al. (B) Deng et al. (C) Xue et al. (D) Milagre et al. (E) Encode. Note that due to differences in library preparation and data analysis it is not appropriate to compare values across datasets.

Supplementary Figure 1

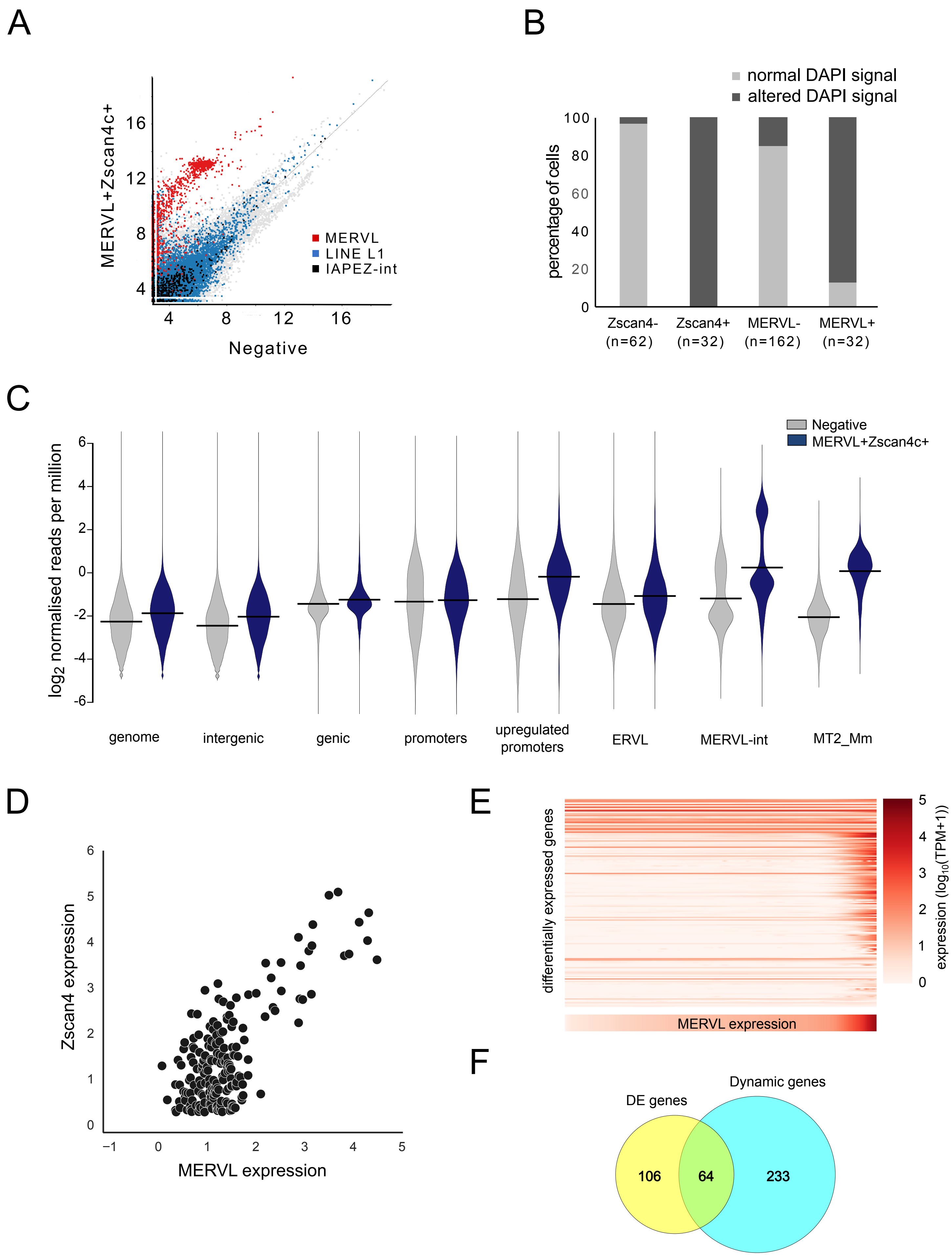

Supplemental Figure 2

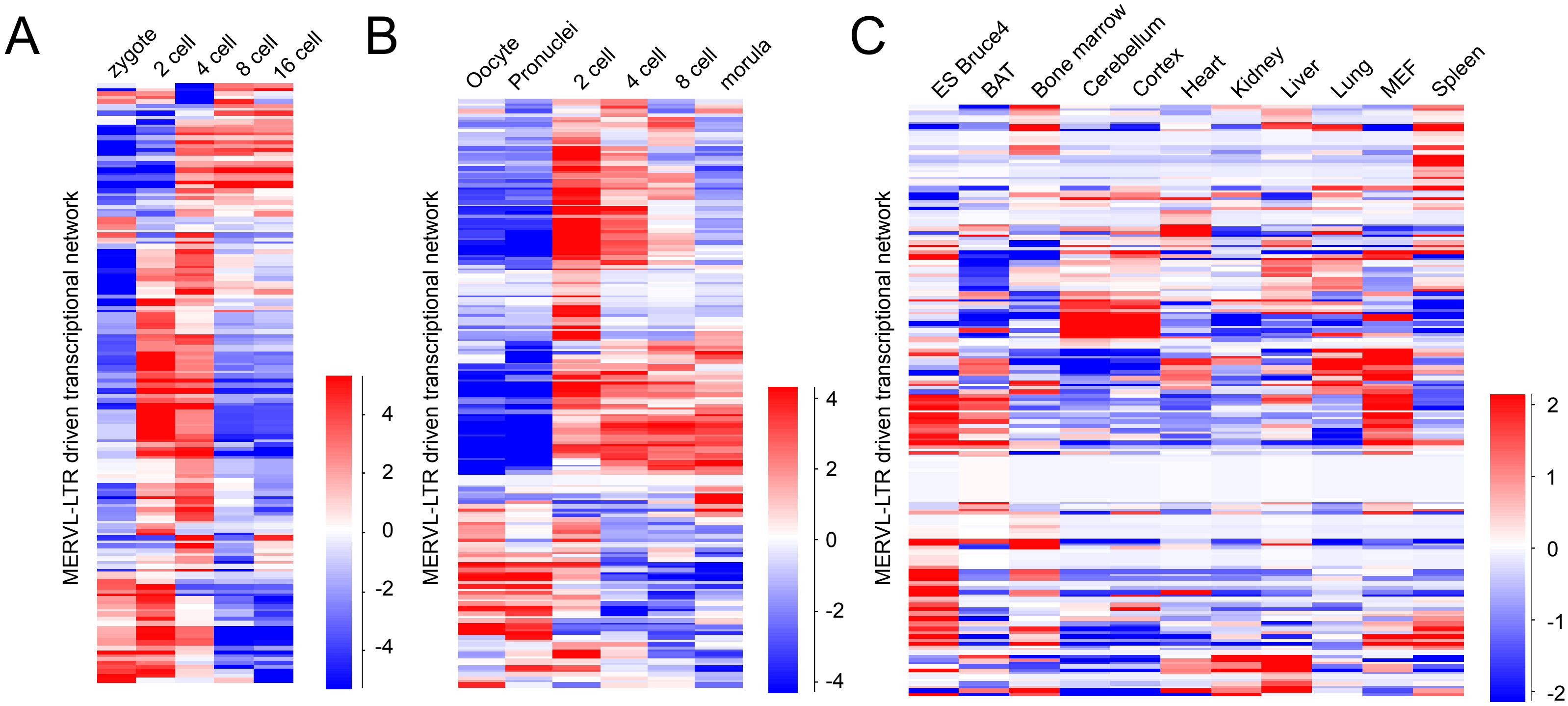

Supplemental Figure 3

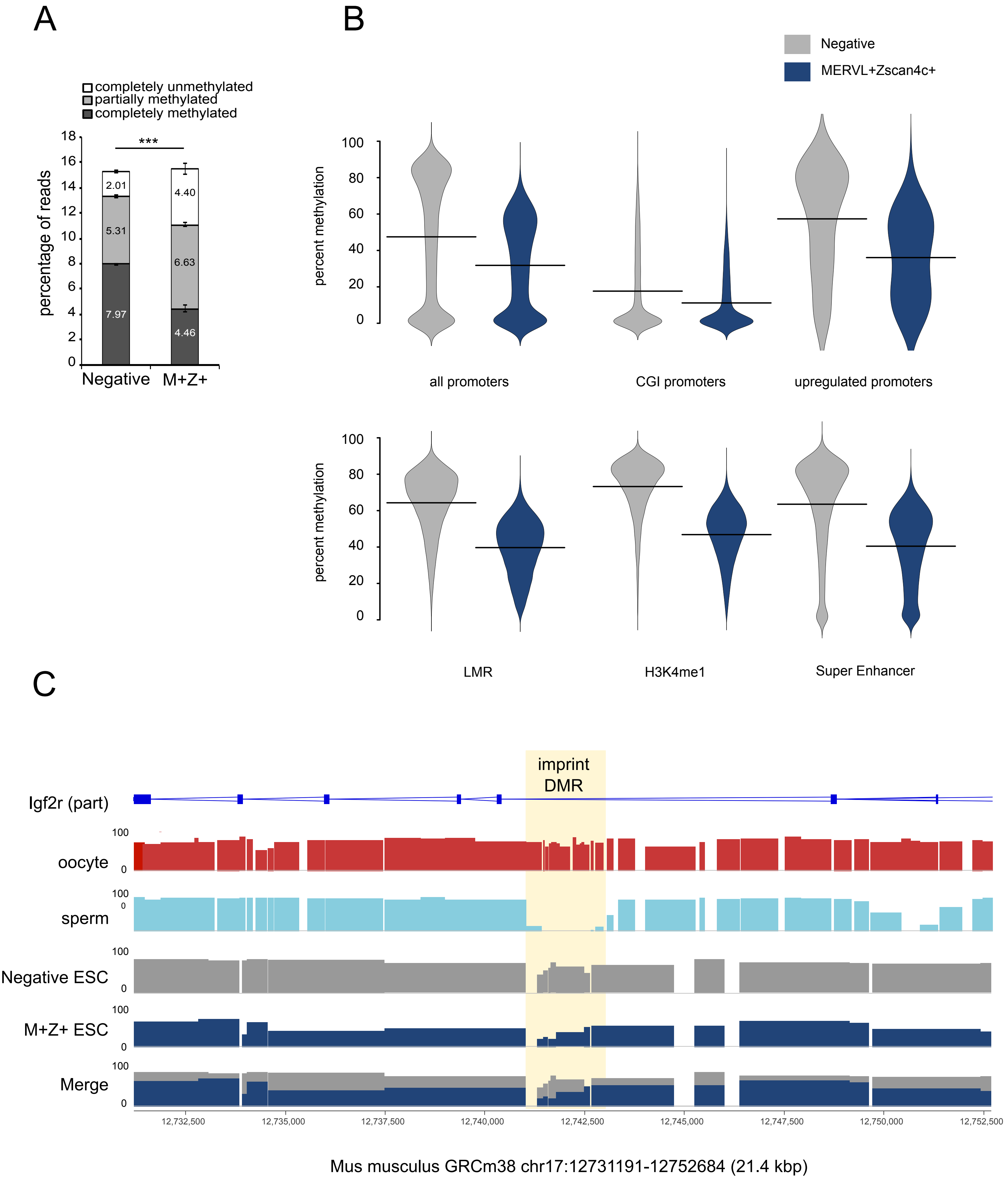

Supplemental Figure 4

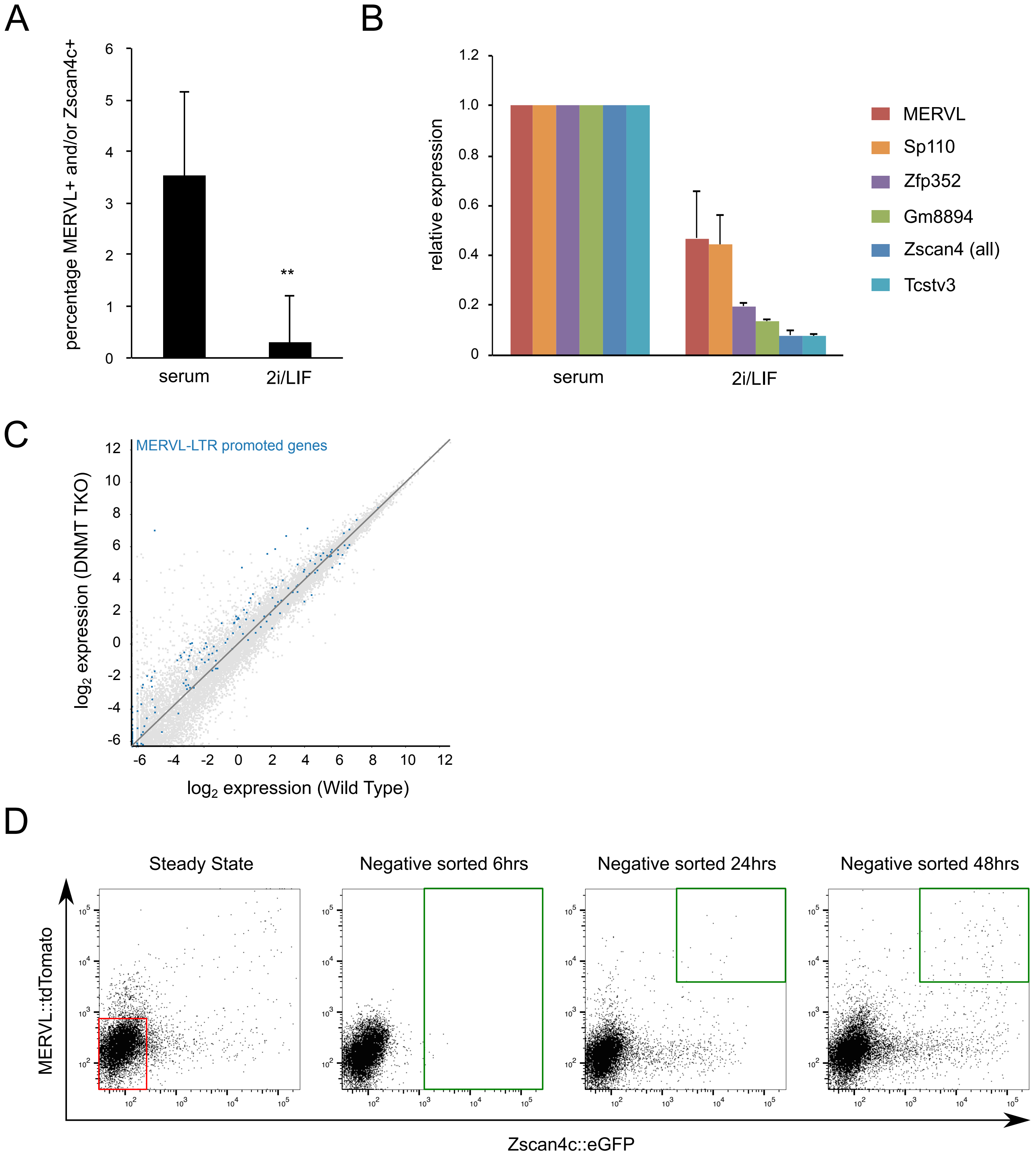

Supplemental Figure 5

A

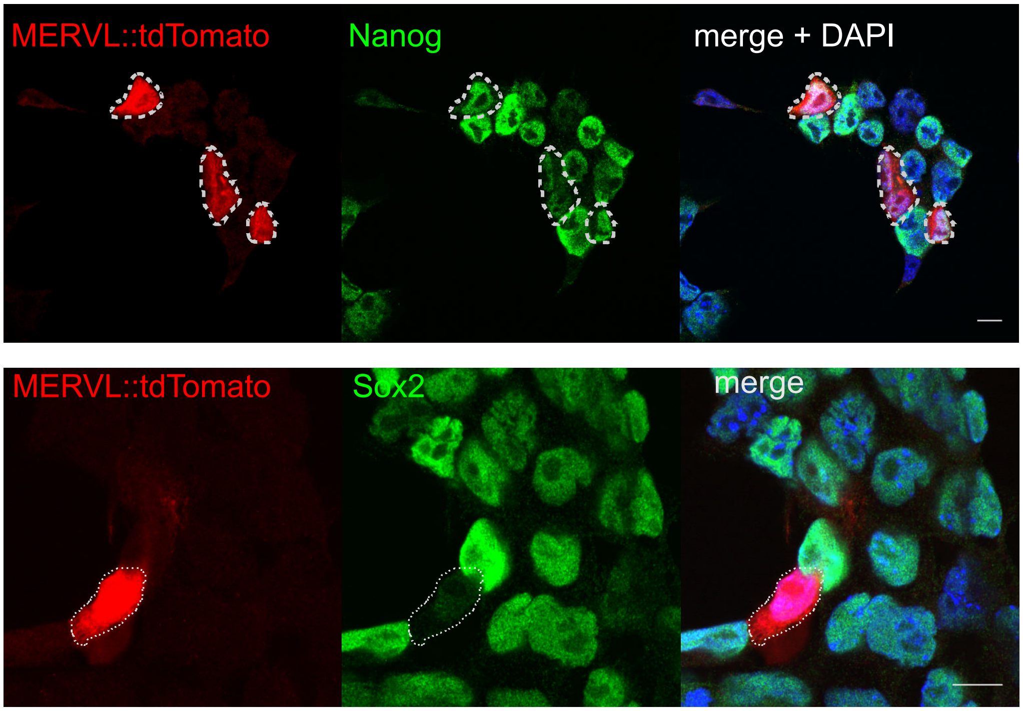

B

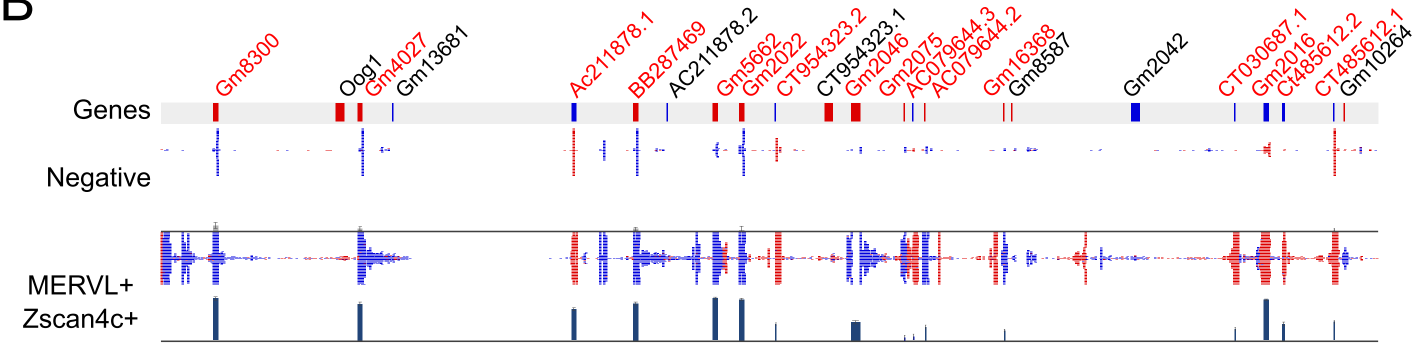

C

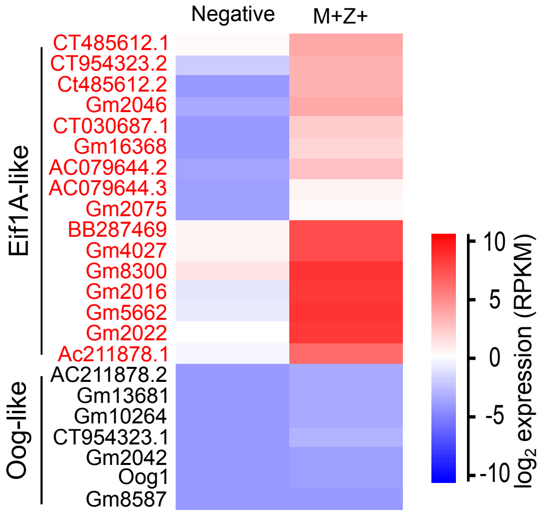

Supplemental Figure 6

A

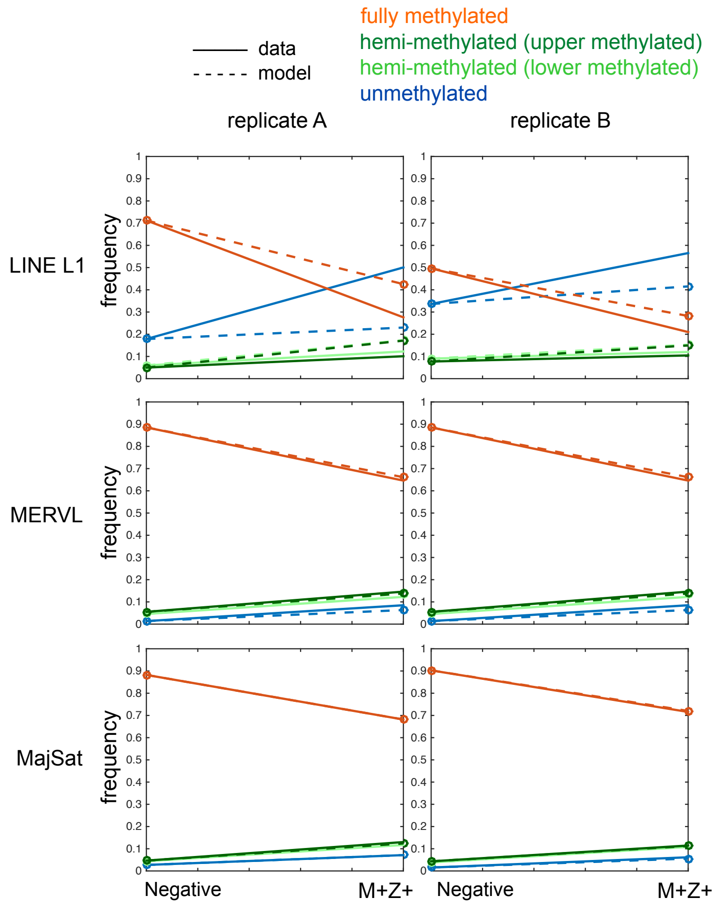

Supplement: Document S1. Supplemental Experimental Procedures and Figures S1–S6 [file mmc1.pdf]
