# Supplementary material for: MERVL/Zscan4 Network Activation Results in Transient Genome-wide DNA Demethylation of mESCs
Source: Cell Rep. 2016 Sep 27;17(1):179–92. doi: 10.1016/j.celrep.2016.08.087 (PMC5055476; doi:10.1016/j.celrep.2016.08.087)

Cell Reports

Article

MERVL/Zscan4 Network Activation Results in Transient Genome-wide DNA Demethylation of mESCs

Graphical Abstract

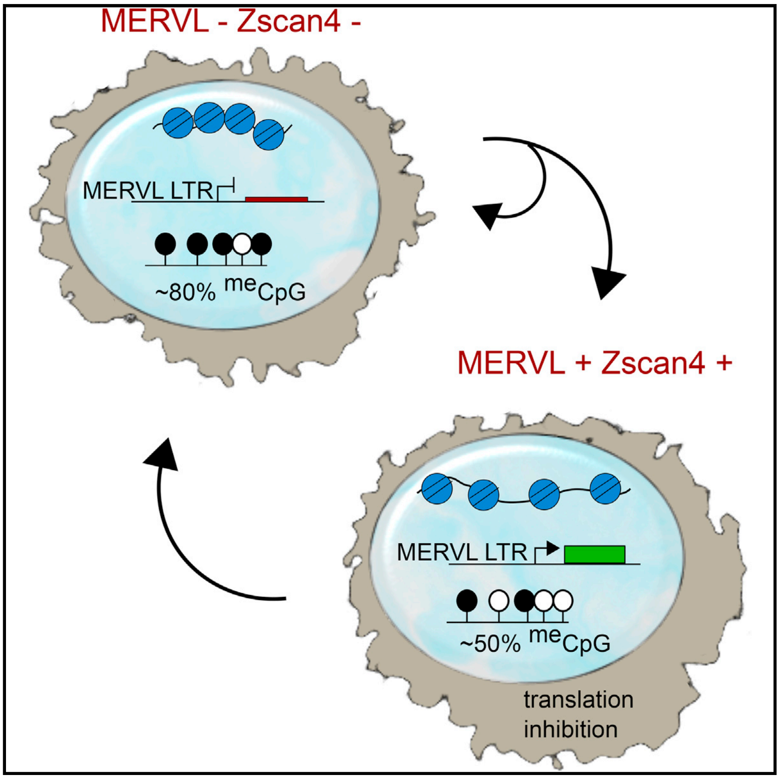

Highlights

- Single-cell transcriptomics reveals dynamics of MERVL/Zscan4 network activation
- MERVL-LTR transcriptional network is expressed in iPSC reprogramming events
- Translation block depletes Dnmt proteins, inducing transient global demethylation
- Passage through the MERVL/Zscan4 state may cause irreversible imprint erasure

Authors

Mélanie A. Eckersley-Maslin, Valentine Svensson, Christel Krueger, ..., Jörn Walter, Sarah A. Teichmann, Wolf Reik

Correspondence

eckersleym@babraham.ac.uk (M.A.E.-M.), wolf.reik@babraham.ac.uk (W.R.)

In Brief

Mouse embryonic stem cells sporadically express preimplantation transcripts, including the MERVL endogenous retrovirus and Zscan4 cluster. Eckersley-Maslin et al. investigate the transcriptional dynamics in these cells and reveal transient genome-wide DNA demethylation accompanying chromatin decompaction. Following state exit, methylation levels are restored, except for genomic imprints, which remain lost.

Accession Numbers

GSE75751
GSE85776
E-MTAB-5058

Eckersley-Maslin et al., 2016, Cell Reports *17*, 179–192
September 27, 2016 © 2016 The Author(s).
http://dx.doi.org/10.1016/j.celrep.2016.08.087

OPEN ACCESS
CellPress

MERVL/Zscan4 Network Activation Results in Transient Genome-wide DNA Demethylation of mESCs

Mélanie A. Eckersley-Maslin,[1,*] Valentine Svensson,[2,3] Christel Krueger,[1] Thomas M. Stubbs,[1] Pascal Giehr,[4] Felix Krueger,[5] Ricardo J. Miragaia,[2,3,6] Charalampos Kyriakopoulos,[7] Rebecca V. Berrens,[1] Inês Milagre,[1] Jörn Walter,[4] Sarah A. Teichmann,[2,3] and Wolf Reik[1,3,8,*]
[1]Epigenetics Programme, Babraham Institute, Cambridge CB22 3AT, UK
[2]EMBL-European Bioinformatics Institute, Wellcome Genome Campus, Hinxton, Cambridge CB10 1SD, UK
[3]Wellcome Trust Sanger Institute, Wellcome Genome Campus, Hinxton, Cambridge CB10 1SA, UK
[4]Laboratory of EpiGenetics, Saarland University, Campus A2 4, 66123 Saarbrücken, Germany
[5]Bioinformatics Group, Babraham Institute, Cambridge CB22 3AQ, UK
[6]Centre of Biological Engineering, University of Minho, Campus de Gualtar, 4710-057 Braga, Portugal
[7]Computer Science Department, Saarland University, Campus E1.3, 66123 Saarbrücken, Germany
[8]Lead Contact
*Correspondence: eckersleym@babraham.ac.uk (M.A.E.-M.), wolf.reik@babraham.ac.uk (W.R.)
http://dx.doi.org/10.1016/j.celrep.2016.08.087

SUMMARY

Mouse embryonic stem cells are dynamic and heterogeneous. For example, rare cells cycle through a state characterized by decondensed chromatin and expression of transcripts, including the Zscan4 cluster and MERVL endogenous retrovirus, which are usually restricted to preimplantation embryos. Here, we further characterize the dynamics and consequences of this transient cell state. Single-cell transcriptomics identified the earliest upregulated transcripts as cells enter the MERVL/Zscan4 state. The MERVL/Zscan4 transcriptional network was also upregulated during induced pluripotent stem cell reprogramming. Genome-wide DNA methylation and chromatin analyses revealed global DNA hypomethylation accompanying increased chromatin accessibility. This transient DNA demethylation was driven by a loss of DNA methyltransferase proteins in the cells and occurred genome-wide. While methylation levels were restored once cells exit this state, genomic imprints remained hypomethylated, demonstrating a potential global and enduring influence of endogenous retroviral activation on the epigenome.

INTRODUCTION

It is well known that mouse embryonic stem cells (ESCs) contain extensive epigenetic and transcriptional heterogeneities, yet the mechanistic details of how cells enter and exit these different states and their importance for stem cell potency and maintenance are only beginning to be understood (reviewed in De Los Angeles et al., 2015; Lee et al., 2014; Torres-Padilla and Chambers, 2014). Intriguingly, a rare group of ESCs express early-embryonic transcripts, including the Zscan4 cluster (Zalzman et al., 2010) and MERVL endogenous retrovirus (Macfarlan et al., 2012). However, the precise molecular dynamics of this process and any potential epigenetic consequences of the endogenous retroviral activation remain unknown.

Transposable elements comprise more than 50% of mammalian genomes and fall into two classes based on the presence or absence of a long-terminal repeat (LTR) (Friedli and Trono, 2015). One class of LTR-retrotransposon (also known as endogenous retroviruses [ERVs]) is the murine endogenous retrovirus with leucine tRNA primer, or MERVL. Expression of MERVL is normally restricted to the two-cell mouse preimplantation embryo but also occurs transiently in rare ESCs (Macfarlan et al., 2012). Similarly, the Zscan4 cluster of zinc-finger proteins, also normally restricted to the two-cell embryo (Falco et al., 2007), is expressed in a subset of ESCs (Zscan4^{+} cells), where it has been proposed to have a role in telomere elongation and genomic stability (Zalzman et al., 2010). While MERVL^{+} cells express Zscan4 transcripts and vice versa (Akiyama et al., 2015; Ishiuchi et al., 2015; Macfarlan et al., 2012), it is unclear if these markers label a single population or overlapping populations of cells with potentially different functions. Furthermore, the dynamics of transcriptional network activation and any epigenetic consequences in these cells remain unclear.

Globally, MERVL^{+} cells have increased levels of acetylated histones (Ishiuchi et al., 2015; Macfarlan et al., 2012) and an increase in chromatin mobility (Bošković et al., 2014; Ishiuchi et al., 2015). Similarly, Zscan4^{+} cells show an increase in H3K27ac, including at repetitive elements such as retrotransposons (Akiyama et al., 2015). Moreover, experimental perturbations that lead to global chromatin changes, including treatment with histone deacetylase (HDAC) inhibitors (Dan et al., 2015; Walter et al., 2016), knockdown of the chromatin assembly factor Caf-1 (Ishiuchi et al., 2015), the chromatin remodeler Chd5 (Hayashi et al., 2016), Hnrnpk (Thompson et al., 2015), or members of repressive chromatin complexes such as Kap-1, Kdm1a, G9a, Hp1, or Rybp (Hisada et al., 2012; Macfarlan

© 2016 The Author(s).
This is an open access article under the CC BY license (http://creativecommons.org/licenses/by/4.0/).

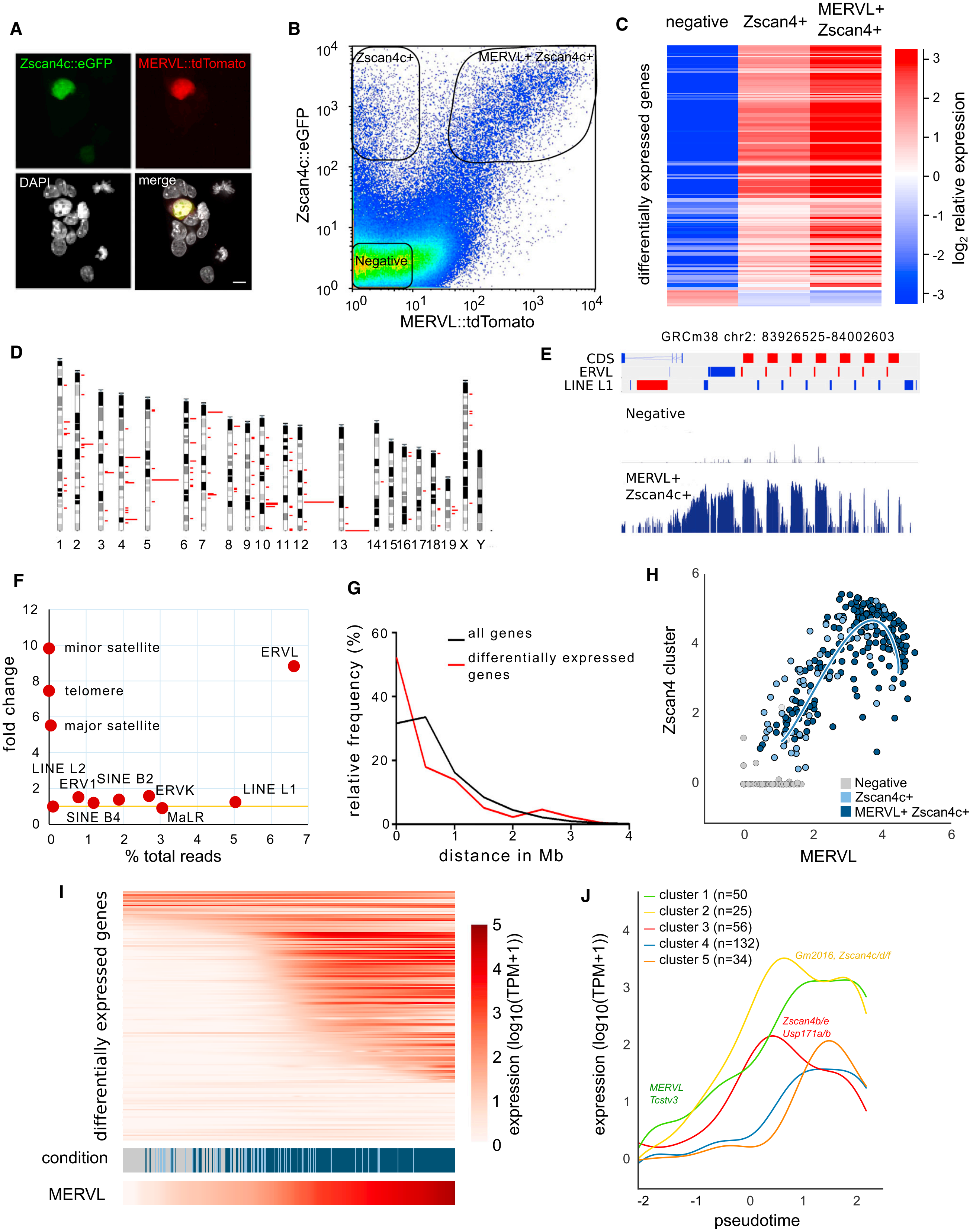

(legend on next page)

et al., 2011, 2012; Maksakova et al., 2013; Rowe et al., 2010), all result in an expansion of MERVL and/or Zscan4 expressing populations (reviewed in Ishiuchi and Torres-Padilla, 2013; Schlesinger and Goff, 2015). Thus, while a link between chromatin decompaction and MERVL/Zscan4 expression is apparent, a comprehensive genome-wide base-resolution analysis of the epigenetic landscape and its dynamics in this transient cell state is lacking.

In this study, we provide a detailed molecular understanding of the transcriptional dynamics and epigenetic characteristics of MERVL$^+$/Zscan4$^+$ cells. Through bulk and single-cell transcriptomics, we observe a coordinated upregulation of a MERVL-LTR-driven transcriptional network that is similarly activated during preimplantation development and induced pluripotent stem cell reprogramming. Interestingly, in MERVL$^+$Zscan4$^+$ cells, inhibition of translation leads to depletion of proteins, including DNA methyltransferases, which in turn results in genome-wide DNA hypomethylation through dilution of methylated cytosines upon DNA replication and cell division. Importantly, while methylation levels are restored following exit from the MERVL$^+$Zscan4$^+$ state, once genomic imprints are lost, they remain demethylated. In this way, ESCs cycling through the MERVL$^+$Zscan4$^+$ state may lead to irretrievable loss of imprints in ESC cultures.

RESULTS

Single-Cell Transcriptomic Analysis of MERVL-LTR-Driven Network Dynamics

The MERVL endogenous retrovirus and other normally developmentally restricted transcripts, including the Zscan4 cluster, are transiently upregulated in a small subset of mouse ESCs (Akiyama et al., 2015; Ishiuchi et al., 2015; Macfarlan et al., 2012; Zalzman et al., 2010), yet the dynamics of their regulation, epigenetic consequences and functional significance in a broader biological context remain poorly understood. In order to explore the molecular regulation of these rare cells, a double reporter ESC line was constructed containing stable transgene integrations of MERVL and Zscan4c promoter-driven tdTomato and EGFP fluorescent reporters, respectively (Figure 1A). These reporters have been previously described to reflect expression patterns of their endogenous counterparts (Macfarlan et al., 2012; Zalzman et al., 2010). While ~1%–2% of cells are labeled by both reporters (MERVL$^+$Zscan4c$^+$), we observed an additional Zscan4c::EGFP$^+$-only population (Figure 1B). However, total RNA sequencing (RNA-seq) revealed the same set of transcripts was upregulated in both single- and double-positive populations (Figure 1C), indicating the reporters are largely interchangeable and mark the same set of ESCs, with the MERVL::tdTomato reporter showing a more restricted expression pattern.

We next defined a set of 172 differentially expressed genes based on the total RNA-seq data (Tables S1 and S2), which was used in all subsequent analyses (see Supplemental Experimental Procedures). Interestingly, many of these genes had no known function and were organized in clusters of tandem repeats (Figures 1D and 1E), suggesting a coordinated and rapid regulation of homologous transcripts. Consistent with previous reports (Akiyama et al., 2015; Ishiuchi et al., 2015; Macfarlan et al., 2012), we observed specific upregulation of MERVL endogenous retroviral elements (Figures 1F and S1A) and found differentially expressed genes to be closer to the MERVL promoter (MT2_Mm) when compared to all genes (Figure 1G). Furthermore, we confirmed and extended by assay for transposase-accessible chromatin sequencing (ATAC-seq) analysis (Figures S1B and S1C) the altered nuclear organization recently described at a global level in MERVL$^+$Zscan4$^+$ cells (Akiyama et al., 2015; Ishiuchi et al., 2015; Macfarlan et al., 2012). The overall increase in chromatin accessibility across the genome was particularly pronounced at promoters of upregulated genes and MERVL elements (MT2_Mm and MERVL-int) (Figure S1C), consistent with the decondensed chromatin structure enabling transcriptional activation of the MERVL MT2_Mm promoter and linked protein-coding genes.

Figure 1. Single-Cell Transcriptomic Analysis of MERVL-LTR Driven Network Dynamics

(A) Representative single z-section through a ESC colony containing a cell co-expressing the Zscan4c::EGFP (top left) and MERVL::tdTomato (top right) reporters. DNA is visualized with DAPI (bottom left). Scale bar, 10 μm.

(B) Flow cytometry plot of the MERVL::tdTomato (x axis) Zscan4c::eGFP (y axis) dual-reporter ESC line. Representative gates used to sort the three populations (negative, Zscan4c$^+$, and Zscan4c$^+$MERVL$^+$) are shown.

(C) Heat map showing relative expression levels of 172 differentially expressed genes in negative (left column), Zscan4$^+$ (middle column) and Zscan4$^+$MERVL$^+$ (right column) sorted populations as determined by total RNA-sequencing (see Supplemental Experimental Procedures). Scale bar depicts $\log_2$ relative expression.

(D) Overview showing localization and frequency of differentially expressed genes (red bars) across the genome.

(E) Schematic overview over the Gm13691 containing cluster on chromosome 2 depicting the organization of coding sequence (CDS), MERVL, and LINE L1 elements. Two data tracks show wiggle plots of total RNA-seq reads for MERVL$^+$Zscan4$^+$ (dark blue) and negative-sorted (gray) cells.

(F) Quantification of total RNA-seq reads mapping to different repeat classes in MERVL$^+$Zscan4c$^+$ and negative-sorted cells, plotted as fold change over percentage of total reads. The yellow line indicates no change.

(G) Relative frequency plot showing distance between gene and nearest MERVL promoter (MT2_Mm) of all genes (black) and differentially expressed genes (red). p value < 0.0001 Mann-Whitney *U* test.

(H) Log_{10} TPM (transcripts per million) values of MERVL (x axis) and the Zscan4 cluster (y axis) of single cells sorted from negative (gray), Zscan4c$^+$ (light blue) and Zscan4c$^+$MERVL$^+$ (dark blue) gates. The solid blue line represents the projected trajectory of the cells in this two-dimensional space, or "pseudotime' (see Supplemental Experimental Procedures).

(I) Smoothed heatmap showing expression of 172 differentially expressed genes (rows) across sorted single cells (columns) ordered by MERVL expression (bottom scale bar). Median Spearman rank correlation was 0.6 between MERVL and differentially expressed genes and −0.08 between MERVL and all genes.

(J) Expression profiles of dynamic clusters of genes across pseudotime, denoting selected genes of interest.

See also Figure S1 and Tables S1, S2, and S3.

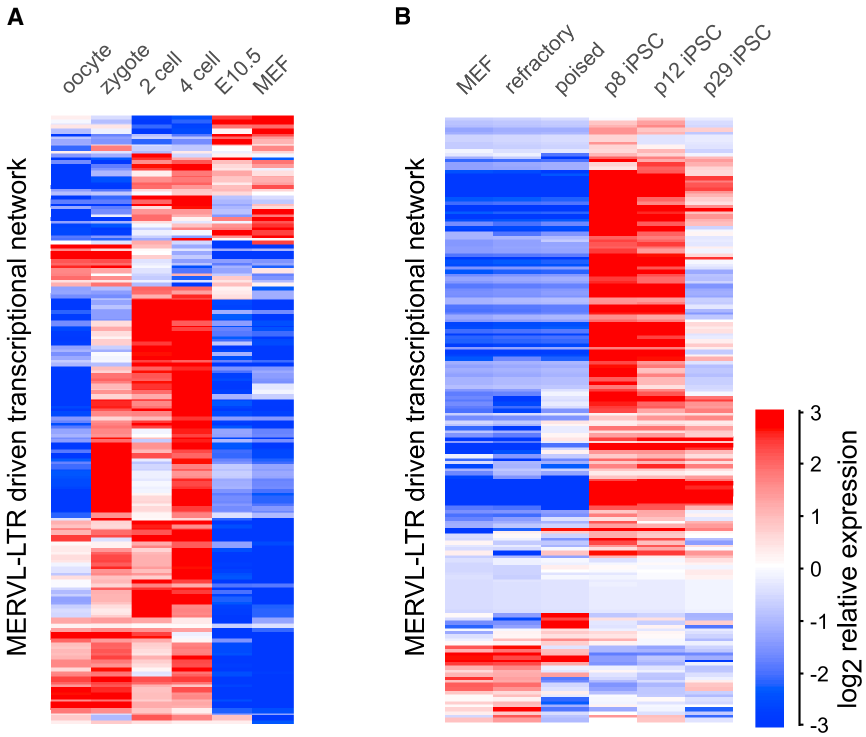

Figure 2. MERVL-LTR-Driven Transcriptional Network Activated upon iPSC Reprogramming

(A and B) Heatmap showing relative probe-normalized expression levels of MERVL$^+$ transcriptional network in early embryos (A) and induced pluripotent stem cell reprogramming (B). MEF, mouse embryonic fibroblasts. Refractory (SSEA1-/Thy1$^+$) and poised (SSEA1$^+$/Thy1$^-$) stages correspond to fluorescence-activated cell sorting (FACS)-sorted cells at day 6, where passage 8 (p8; corresponding to day 21), p12 (corresponding to day 29) iPSCs represent intermediate-late stages of reprogramming and p29 (corresponding to day 60) iPSCs are fully reprogrammed. Scale bar depicts $\log_2$ relative expression. Data are from Park et al. (2015) (A) and (I.M., unpublished data) (B).

See also Figure S2 and Table S4.

To further understand the dynamics of MERVL-LTR-driven gene activation, single-cell RNA-sequencing was performed on 319 cells sorted from the negative (75 cells), Zscan4c$^+$ only (52 cells), and MERVL$^+$Zscan4c$^+$ (192 cells) populations (Figure 1B). The endogenous counterparts of the reporters were co-regulated across the single cells (Figure 1H), in that MERVL$^+$ cells expressed the Zscan4 cluster and vice versa. There was a synchronous graded upregulation of the differentially expressed genes across the single cells (Figure 1I). Notably, cells from the Zscan4c$^+$ only and MERVL$^+$Zscan4c$^+$ sorted fractions were intermingled, suggesting that the two separate populations seen by flow cytometry represented a difference in the kinetics and/or strength of the reporters and not true distinct populations. Importantly, our findings were independent of the reporters and sorting strategies used, as re-analysis of published unsorted ESC single-cell datasets (Buettner et al., 2015; Kolodziejczyk et al., 2015; Kumar et al., 2014) also revealed a similar coordinated and gradual upregulation of the MERVL-LTR-promoted transcriptional network (Figures S1D and S1E).

Next, the transcriptional dynamics of these transient cells was analyzed. By creating a "pseudotime" trajectory, cells were ordered as they activated the MERVL-LTR-driven transcriptional network (Figure 1H; see Supplemental Experimental Procedures for details). This permitted subsequent identification of genes that were dynamic over pseudotime. These genes largely overlapped with the differentially expressed genes identified above (Figure S1F) and fell into five clusters (Figure 1J; Table S3). Interestingly, the first cluster to be activated (cluster 1) includes MERVL, whereas clusters 2 and 3, which contain the Zscan4 genes, were activated soon after. Intriguingly, clusters 4 and 5 became expressed as clusters 1 and 2 began to decrease, suggesting they may include potential negative regulators of the MERVL-LTR-promoted transcriptional network. In summary, the unbiased inference of the ordering of these cells based on modeling of the single cell RNA-sequencing (scRNA-seq) data allowed us to make predictions about the transcriptional network dynamics of MERVL$^+$Zscan4$^+$ cell regulation.

MERVL-LTR-Driven Transcriptional Network Activated upon iPSC Reprogramming

The MERVL-driven transcriptional network that is upregulated in MERVL$^+$Zscan4$^+$ cells is similarly activated upon zygotic genome activation in mouse preimplantation embryos (Ishiuchi et al., 2015; Kigami et al., 2003; Macfarlan et al., 2012) (Figures 2A, S2A, and S2B; Table S4). This coincides with a developmental time window in which dynamic chromatin remodeling events accompany changes in cellular identity and potency. We therefore investigated whether the MERVL-LTR-driven transcriptional network is similarly upregulated during another cellular reprogramming event, specifically during induced pluripotent stem cell (iPSC) reprogramming. Strikingly, while there was no upregulation of the MERVL-LTR-driven transcriptional network at early stages of iPSC reprogramming, consistent with previous reports (Friedli et al., 2014), we observed a dramatic and transient upregulation of the MERVL-LTR-driven transcriptional network in the intermediate-late stages of iPSC reprogramming (Figure 2B; Table S4). In contrast, no upregulation of the MERVL-LTR driven transcriptional network was found in somatic tissues (Figure S2C; Table S4). Therefore, MERVL-LTR transcriptional network activation occurs not only in two-cell embryos *in vivo* but also *in vitro* in a subset of mouse ESCs and during iPSC reprogramming.

MERVL^{+}Zscan4^{+} Cells Undergo Global DNA Demethylation

One commonality in the programs that transiently upregulate the MERVL-LTR-driven transcriptional network is that they coincide with global changes in DNA methylation landscapes. We therefore investigated the methylomes of MERVL^{+}Zscan4^{+} cells. Mass spectrometry revealed a decrease in 5-methylcytosine (Figure 3A) and increase in 5-hydroxymethylcytosine (Figure 3B) in MERVL^{+}Zscan4c^{+} sorted cells. This was confirmed by whole-genome bisulfite sequencing, which revealed a substantial decline in overall CpG methylation from 80% to 56% (Figures 3C and S3A).

The decrease in CpG methylation occurs genome-wide and is not restricted to any individual genomic feature. There was an overall shift in the methylation levels of probes between MERVL^{+}Zscan4c^{+} and negative-sorted cells (Figure 3D) that was evenly distributed across chromosomes (Figure 3E) and not specific to differentially expressed regions. Methylation levels were reduced across all genomic locations and features analyzed, including gene bodies, promoters, enhancer regions, and all repeat classes including MERVL elements (Figures 3F, 3G, and S3B). Intriguingly, genomic imprints also showed reduced methylation levels (Figures 3F and S3C). In summary, MERVL^{+}Zscan4^{+} cells have a global loss of DNA methylation across all genomic contexts unlinked to the transcriptional changes occurring in the cell.

Acute DNA Demethylation Is Not Sufficient for MERVL Network Activation

Next, we investigated whether the global DNA demethylation observed was the cause of the MERVL network activation or a consequence of being in this state. First, global DNA demethylation was induced in ESCs using an inducible Dnmt1 knockout line (Sharif et al., 2016). Within 3 days of Dnmt1 loss, CpG methylation levels genome-wide (Figure 4A) and at MERVL elements (Figure 4B) were reduced to ~30%, which is less than what is seen in MERVL^{+}Zscan4^{+} cells. Despite this, there was no activation of the MERVL endogenous retrovirus (Figure 4C) or MERVL-LTR transcriptional network (Figure 4D), suggesting that acute DNA demethylation during this time window is not sufficient to enter the MERVL^{+}Zscan4^{+} state. Similarly, there was no upregulation of the MERVL-LTR transcriptional network during or following the global DNA demethylation that is observed when switching ESCs from serum containing media to naive 2i conditions (Figure 4E) (Ficz et al., 2013; Habibi et al., 2013; Leitch et al., 2013; von Meyenn et al., 2016). Instead, the proportion of MERVL^{+}Zscan4^{+} cells is reduced upon long-term naive 2i culture (Figures S4A and S4B) as previously reported (Macfarlan et al., 2012). Furthermore, the MERVL-LTR transcriptional network is not significantly upregulated in DNMT triple-knockout (TKO) cells (Domcke et al., 2015) that are devoid of DNA methylation (Figure S4C), again supporting the notion that MERVL/Zscan4 network activation is not a consequence of global DNA demethylation.

Next, we followed the kinetics of DNA demethylation and MERVL-LTR-driven network activation to further understand the causal relationship between the two. If DNA demethylation followed MERVL^{+}Zscan4^{+} state activation, the longer a cell remains in this state, the more demethylated it would become. Negative cells were isolated by flow cytometry and returned to culture, allowing the newly arising MERVL^{+}Zscan4c^{+} cells to be identified (Figure 4F). By 72 hr, the initial negative sorted cells had re-established the steady-state proportion of MERVL^{+}Zscan4c^{+} cells (Figure S4D). Importantly, during this time, there was a progressive loss of DNA methylation (Figure 4G). Therefore, global DNA demethylation occurs subsequently to MERVL-LTR transcriptional network activation and is not sufficient to induce the MERVL^{+}Zscan4^{+} state.

Translation Block Leads to Depletion of Dnmt Enzymes

Next, the mechanism resulting in global DNA demethylation was investigated. mRNA levels of the DNA methylation machinery enzymes were mostly similar in the MERVL^{+}Zscan4c^{+} cells compared to negative-sorted control cells (Figure 5A). Despite this, immunofluorescence staining of Zscan4c^{+} cells revealed a dramatic reduction in the protein levels of maintenance methyltransferase Dnmt1 and de novo methyltransferases Dnmt3a and Dnmt3b (Figures 5B and 5C), with some Zscan4c^{+} cells showing complete absence of protein.

This uncoupling of the transcriptome and proteome is not limited to the Dnmt enzymes. Despite similar transcript levels (Figure 5D), immunofluorescence analysis revealed a complete absence of Oct4 and Sox2 and reduced Nanog protein levels in MERVL^{+} cells (Figures 5E and S5A), consistent with previous reports (Ishiuchi et al., 2015; Macfarlan et al., 2012). This suggests that a general rather than targeted method of protein depletion occurs in MERVL^{+}Zscan4^{+} cells. Indeed, global repression of nascent protein synthesis in Zscan4^{+} cells has been previously reported and proposed to be driven by a cluster of genes on chromosome 2 (mm10 Chr12:87473449-88356013) with high sequence homology to eukaryotic initiation factor 1a (Eif1a) (Hung et al., 2013). We confirmed both an inhibition of active protein synthesis in MERVL^{+}Zscan4c^{+} cells (Figures 5F and 5G) as well as upregulation of the Eif1A-like cluster (Figures S5B and S5C). Together, this supports the notion that loss of Dnmt and pluripotency proteins in MERVL^{+}Zscan4^{+} cells is due to translation inhibition.

Loss of Dnmt Activity Is Sufficient for Global DNA Demethylation

Our results suggest that the global reduction in DNA methylation was due to impaired DNA methyltransferase activity resulting directly from the loss of the proteins in these cells. To analyze the relative contributions of different Dnmts, we performed hairpin bisulfite analysis which retains linkage of top and bottom strands of individual DNA duplexes. CpGs for LINE L1, MERVL, and major satellites were classified as fully methylated, fully unmethylated, or hemimethylated (in which the top strand was methylated and bottom unmethylated or *vice versa*). As expected, there was a decrease in fully methylated duplexes (Figure 6A), consistent with the global loss of DNA methylation. This corresponded to an increase in both unmethylated and hemimethylated molecules.

To predict the relative efficiencies of the maintenance and *de novo* methyltransferases, a hidden Markov model (Arand et al., 2012) was run on the hairpin bisulfite data (Figure S6A). This

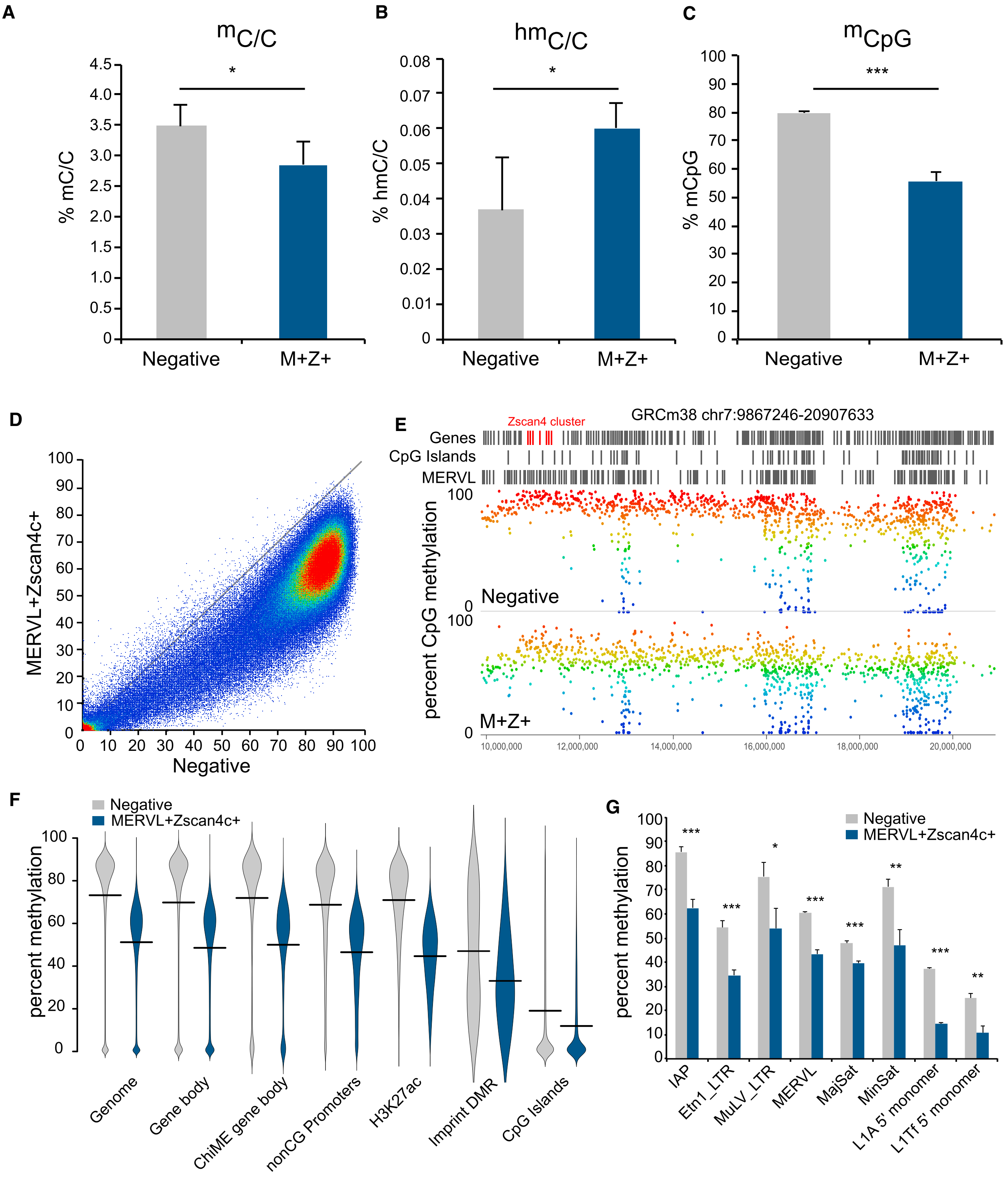

Figure 3. MERVL⁺Zscan4⁺ Cells Undergo Global DNA Demethylation

(A and B) Total levels of (A) 5-methylcytosine and (B) 5-hydroxymethylcytosine as a percentage of total cytosine in negative-sorted (gray) and MERVL$^+$Zscan4c$^+$ (M$^+$Z$^+$, blue) cells as determined by mass spectrometry. Bars represent mean + SD of three biological replicates. *5mC p value = 0.015, *5hmC p value = 0.034, two-tailed paired t test.

(legend continued on next page)

enabled the specific methylation efficiencies for the different Dnmts to be calculated based on the experimental data. For all regions, the model predicted a complete absence of any *de novo* activity (Dnmt3a/3b) and a reduced maintenance (Dnmt1) efficiency of 55%–76% compared to negative-sorted cells (Figure 6B). Importantly, MERVL$^+$Zscan4c$^+$ cells exist in all stages of the cell cycle (Figure 6C), albeit with a prolonged G2/M phase (Fujii et al., 2015; Storm et al., 2014), and undergo mitosis (Figure 6D), permitting a replication-dependent mechanism of demethylation. Therefore, the global DNA demethylation is likely a direct result of cell division with reduced maintenance and *de novo* DNA methylation (Figure 6E).

Epigenetic Consequences of Passing through the MERVL$^+$Zscan4$^+$ State

Lastly, the epigenetic and functional consequences of the MERVL$^+$Zscan4$^+$ state were investigated. Cells were isolated by flow cytometry using the Zscan4c and MERVL reporters and then placed back in culture (Figure 7A). By 72 hr, the cells had mostly exited the MERVL$^+$Zscan4c$^+$ state and returned to the negative population (Figures 7B and 7C), confirming that cells are able to cycle out of the state. Immunofluorescence staining revealed the newly emerging negative cells now expressed pluripotency and Dnmt proteins (Figure 7D), indicating that the translation block had been lifted and protein synthesis resumed. We next assessed whether the methylome of these cells was restored. MERVL$^+$Zscan4c$^+$ cells were sorted and replated and the newly emerging negative cell population isolated for bisulfite analysis. Within 3 days, methylation levels of the cells that had now left the MERVL$^+$Zscan4c$^+$ state had returned to steady-state levels (Figure 7E), confirming that the demethylation observed in these cells is a transient event and does not persist once the cells exit the state.

Finally, the functional consequences of passing through the MERVL$^+$Zscan4$^+$ state were assessed. In particular we investigated the methylation status of imprinted regions: clusters of genes expressed exclusively from either the maternal or paternal alleles and controlled through differential methylation of imprint control regions or differentially methylated regions (DMRs). Loss of imprinting is associated with severe developmental disorders and cancer progression, and thus, imprints must be carefully maintained (reviewed in Barlow and Bartolomei, 2014). Significantly, in MERVL$^+$Zscan4c$^+$ cells, the global DNA demethylation extended to the imprint DMRs (Figures 3F and 7F). Thus, we investigated whether this imprint loss endured in cells that had passed through the MERVL$^+$Zscan4$^+$ state by further analysis of the newly emerging negative cells (see above). Consistent with a restoration of DNA methylation, CpG methylation levels genome-wide and at gene bodies returned to steady-state negative levels by 7 days (Figure 7F). Strikingly, imprint DMRs remained hypomethylated in the cells that had passed through the MERVL$^+$Zscan4c$^+$ state (Figure 7F). To further confirm this, negative cells and MERVL$^+$Zscan4c$^+$ cells were sorted by flow cytometry and then returned to culture for 14 days, after which bulk populations were collected. Imprint DMRs were amplified from bisulfite-treated DNA and sequenced, yielding >1,000-fold coverage per region analyzed. As expected, we observed intermediate methylation levels in negative-sorted cells resulting from a mix of methylated and unmethylated alleles (Figure 7G). However, the DMRs examined of cells that had passed through the MERVL$^+$Zscan4c$^+$ state were hypomethylated compared to controls (Figures 7G and 7H), implying that once the imprints are lost, they are not restored.

In summary, through a combination of single-cell transcriptome and genome-wide epigenetic analyses, we provide significant advances towards our understanding of MERVL$^+$Zscan4$^+$ cells. We reveal a graded upregulation of a MERVL-promoter-driven transcriptional program as cells enter the MERVL$^+$Zscan4$^+$ state, which is similarly upregulated in the two-cell embryo and during iPSC reprogramming. Intriguingly, cells that upregulate the MERVL-LTR-promoted transcriptional network exhibit genome-wide DNA demethylation caused by a translation-block-induced depletion of Dnmt proteins in the cells. While DNA methylation is regained genome-wide once the cell exits the state, genomic imprints are not restored, providing a potential mechanism of how imprint erasure may occur in ESC cultures.

DISCUSSION

We have provided insights into the dynamics and functional consequences of a MERVL-LTR-driven transcriptional network in mouse ESCs. Activation of this network coincides with dramatic chromatin remodeling and ultimately results in genome-wide DNA demethylation. The following model for MERVL network activation in ESCs is proposed (Figure 7I): Chromatin decompaction enables transcription factor accessibility to otherwise heterochromatic and inaccessible MERVL MT2_Mm promoters (Akiyama et al., 2015; Ishiuchi et al., 2015). This drives transcription of full-length MERVL elements and/or downstream

(C) Percentage of total CpG methylation determined by whole-genome bisulfite sequencing in negative-sorted (gray) and MERVL$^+$Zscan4c$^+$ (M$^+$Z$^+$, blue) cells. Bars represent mean + SD of three biological replicates. Difference is statistically significant (***p = 0.000188, homoscedastic two-tailed t test).

(D) Scatterplot comparing percentage of methylated cytosines between negative-sorted (x axis) and MERVL$^+$Zscan4c$^+$ (y axis) cells. Methylated cytosines were counted for each rolling 50-CpG window genome-wide and are expressed as percent of total cytosines per window. R = 0.469.

(E) CpG methylation across part of chromosome 7 for negative-sorted (top track) and MERVL$^+$Zscan4c$^+$ (M$^+$Z$^+$) cells (bottom track). CpG methylation was quantified for each 50-CpG window and is shown as percentage of total CpGs per window. Color scale ranges from 0% methylated (blue) to 100% methylated (red). Top annotation track shows location of genes (gray) with the Zscan4 cluster highlighted in red; the middle annotation track shows location of CpG islands and bottom annotation track position of annotated MERVL elements.

(F) Bean plots showing distribution of methylation levels for different genome features between negative-sorted (gray) and MERVL$^+$Zscan4c$^+$ (blue) cells. Lines represent mean values.

(G) Methylation levels of different repeat classes between negative-sorted (gray) and MERVL$^+$Zscan4c$^+$ (blue) cells. Bars represent means + SD of three biological replicates.

See also Figure S3.

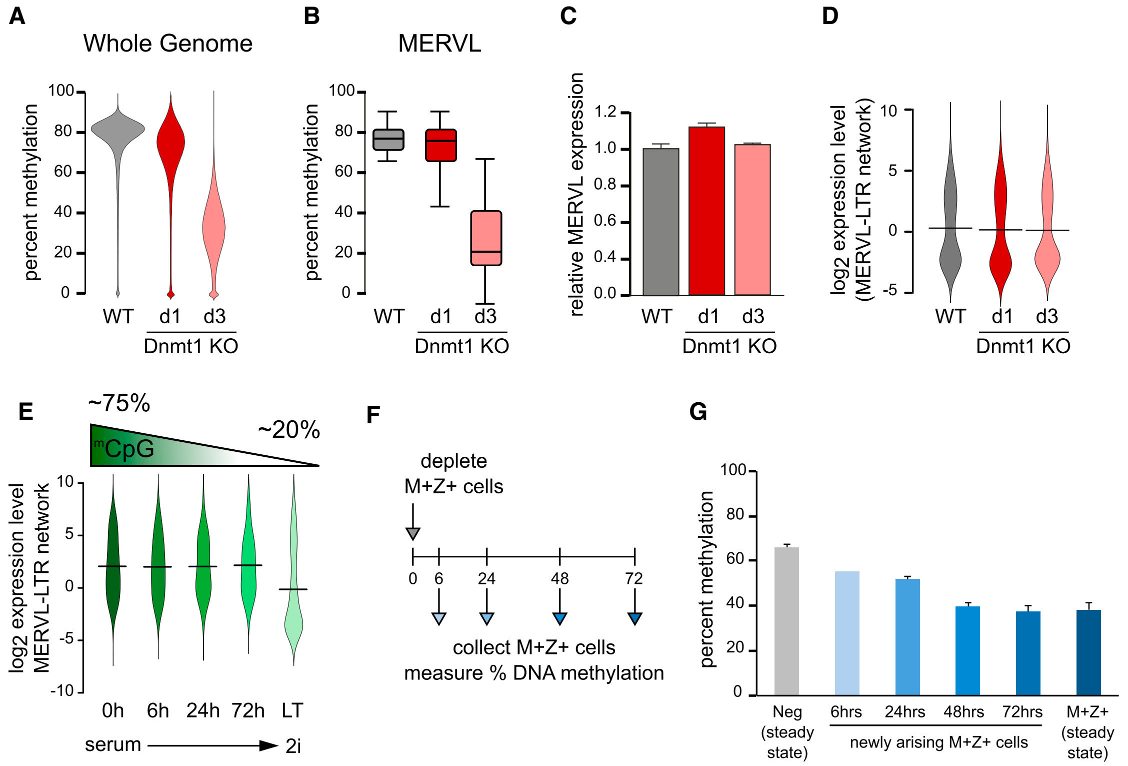

Figure 4. Acute DNA Demethylation Is Not Sufficient for MERVL Network Activation

(A and B) Genome-wide (A) and MERVL (B) CpG methylation levels measured by bisulfite sequencing of wild-type (gray) and conditional Dnmt1 knockout ESCs induced for 1 day (dark red) or 3 days (pink).

(C) qRT-PCR analysis of MERVL expression in wild-type (gray) and conditional Dnmt1 knockout ESCs induced for 1 day (dark red) or 3 days (pink). Bars represent mean + SD of three biological replicates.

(D) Bean plots showing $\log_2$ expression levels of the MERVL-LTR-driven transcriptional network in wild-type (gray) and conditional Dnmt1 knockout ESCs induced for 1 day (dark red) or 3 days (pink).

(E) Bean plots showing $\log_2$ expression levels of the MERVL-LTR-driven transcriptional network in serum-treated (0 hr, dark green), and cells cultured in naive 2i conditions for 6 hr, 24 hr, 72 hr or long-term (LT; light green). Data were reanalyzed from von Meyenn et al. (2016). Average methylation levels are depicted above.

(F) Schematic showing release experiment in which MERVL$^+$Zscan4c$^+$ (M$^+$Z$^+$) cells are depleted from the steady-state population by flow cytometry sorting of the negative gate, followed by re-culturing. The "new" MERVL$^+$Zscan4c$^+$ (M$^+$Z$^+$) cells were subsequently collected by flow cytometry at the indicated time points for methylation analysis.

(G) Genome-wide methylation levels determined by PBAT analysis. Steady-state negative-sorted (light gray, first column) and MERVL$^+$Zscan4c$^+$ (M$^+$Z$^+$) sorted (dark blue, last column) cells are shown for reference. The other columns (light to dark blue) reflect cells that were initially sorted as negative, returned to culture for 6, 24, 48, or 72 hr, and then re-sorted as MERVL$^+$Zscan4c$^+$ (M$^+$Z$^+$) positive. Bars represent average of three biological replicates of 100 cells each ± SD, except the 6-hr time point (n = 1).

See also Figure S4.

sequences, including clusters of tightly packed repeated transcripts of mostly unknown function. Among these are the Eif1a-like family members that may act as dominant-negative inhibitors of translation, causing a depletion of proteins in the cell. This includes DNA methyltransferases, whose loss results in a reduced ability to establish and maintain DNA methylation following DNA replication and consequently global loss of DNA methylation genome-wide. Crucially, while genome-wide methylation levels are restored following exit from the MERVL$^+$Zscan4$^+$ state, genomic imprints are not. Not all systems of extensive DNA demethylation result in imprint erasure; while ESCs undergo demethylation in 2i conditions, imprints are usually protected (Ficz et al., 2013; Habibi et al., 2013). However, addition of vitamin C to 2i conditions has recently been shown to further demethylate the genome, including imprints (Walter et al., 2016).

Variable imprint loss frequently occurs in mouse ESC lines and ESC-derived fetuses (Dean et al., 1998; Deng et al., 2007; Humpherys et al., 2001; Sun et al., 2012). In our study, we demonstrate that the global DNA demethylation observed in MERVL$^+$Zscan4$^+$ cells extends to genomic imprints. While genome methylation levels are restored when cells exit the MERVL$^+$Zscan4$^+$ state, the failure to restore genomic imprints provides a potential mechanism of imprint loss in mouse ESCs. Consistently, mouse ESCs (which have the MERVL$^+$ Zscan4$^+$ state) are more vulnerable to imprint erasure than epiblast stem cells (EpiSCs) (Sun et al., 2012). For genome-wide and imprint DNA demethylation to occur, a cell must

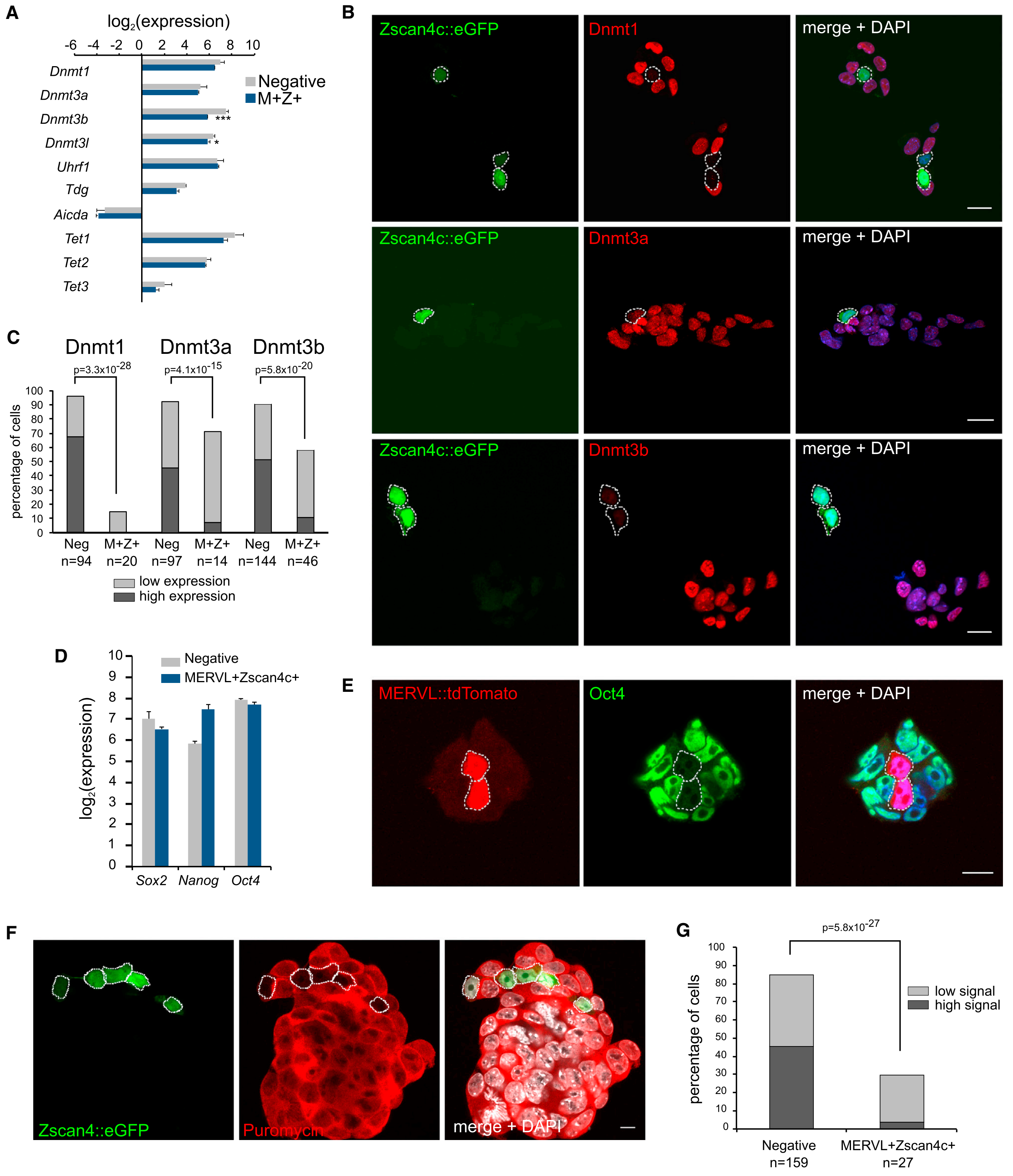

Figure 5. Translation Block Leads to Depletion of Dnmt Enzymes

(A) Expression levels of DNA methylation machinery transcripts between negative-sorted (gray) and MERVL$^+$Zscan4c$^+$ (M$^+$Z$^+$, dark blue) cells as determined by RNA-sequencing. Bars represent mean + SD of at least three biological replicates. *$p < 0.05$, ***$p < 0.0001$, homoscedastic two-tailed t test.

(B) Representative single z-slices of cells labeled by Zscan4c::EGFP reporter (green) immunostained for Dnmt1 (top row, red), Dnmt3a (middle row, red), or Dnmt3b (bottom row, red). Blue depicts DAPI staining. Zscan4c$^+$ cells are outlined in white dotted lines. Scale bar represents 25 μm.

(legend continued on next page)

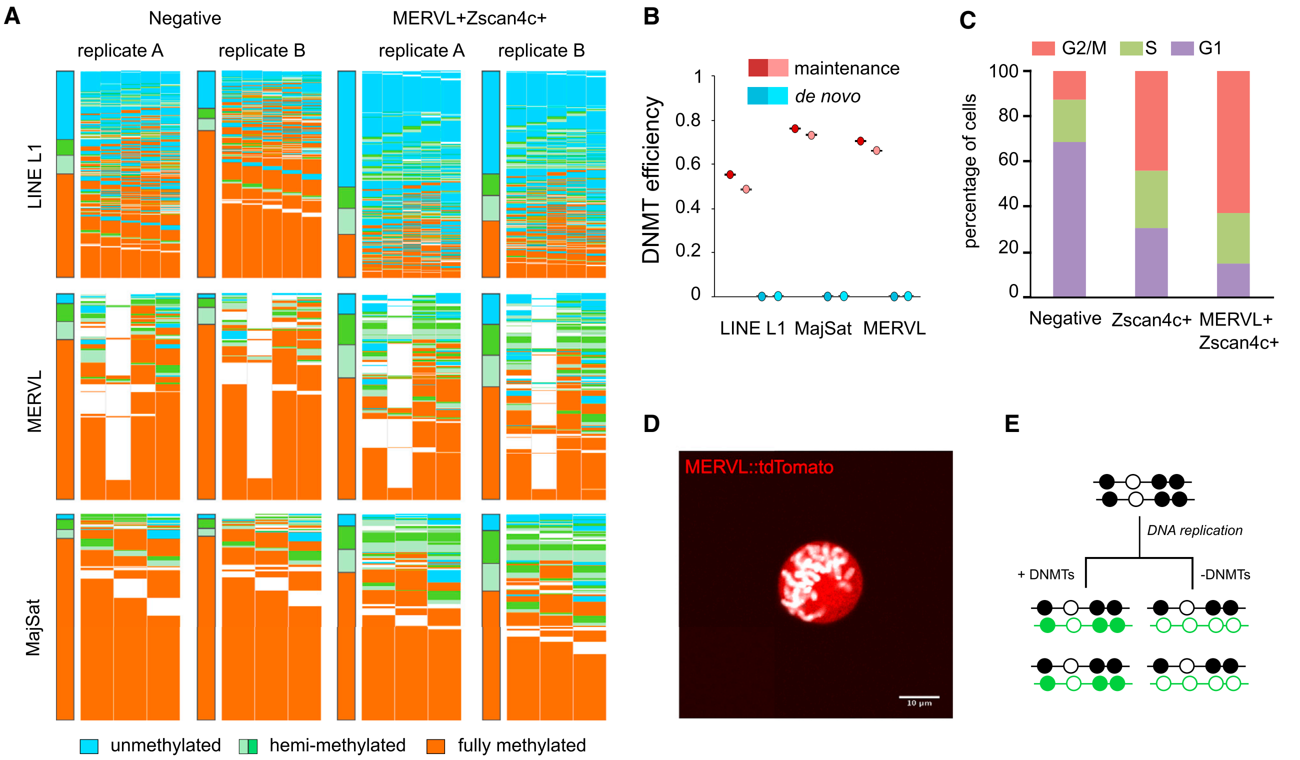

Figure 6. Loss of Dnmt Activity Sufficient for Global DNA Demethylation

(A) Hairpin bisulfite analysis of LINE L1 (top), MERVL (middle), or major satellites (bottom) elements in two biological replicates of negative-sorted (first two columns) or MERVL$^+$Zscan4c$^+$ (last two columns) cells. Each row represents an individual read that was classified as fully methylated (orange), hemimethylated on top (light green) or bottom (dark green) strands, or fully unmethylated (blue).

(B) Predicted activity of maintenance (red) and *de novo* (blue) Dnmt enzymes for the three repeat classes. Color shades represent biological replicates; error bars represent the error of the model.

(C) Cell-cycle distribution of cells into G2/M (red), S (green) or G1 (purple) phases, determined by single-cell RNA-sequencing analysis as previously described (Scialdone et al., 2015).

(D) Single z-slice confocal image of a mitotic cell expressing the MERVL::tdTomato reporter (red), demonstrating the ability of MERVL$^+$Zscan4c$^+$ cells to undergo mitosis either during or following MERVL transcriptional activation. DAPI is shown in white. Scale bar represents 10 μm.

(E) Schematic showing methylation dynamics following replication with (left) and without (right) activity of Dnmt enzymes. Filled circles represent methylated CpGs, and empty circles represent unmethylated CpGs. Newly synthesized DNA strands are depicted in green.

See also Figure S6.

undergo DNA replication while in the MERVL$^+$Zscan4$^+$ state. Given that ~1% of ESCs are in the MERVL$^+$Zscan4$^+$ state at a given time, of which ~20% are in S phase, the actual manifestation of imprint loss through this mechanism would be infrequent and sporadic, and not all ESC cultures will lose imprints in this way. Increasing the frequency of entering the MERVL$^+$ Zscan4$^+$ state and/or clonal expansion would increase the risk of imprint erasure, although we cannot rule out the contribution of a stress response or that other mechanisms contribute towards imprint loss. This has important consequences if ESCs

(C) Semiquantitative analysis of immunofluorescence staining of protein levels. Cells were scored as having high (dark), low (light), or no expression of Dnmt1, Dnmt3a, or Dnmt3b. Cells were subsequently scored using either the Zscan4c::EGFP or MERVL::tdTomato reporters as negative or MERVL$^+$Zscan4c$^+$ (M$^+$Z$^+$) cells. Differences are statistically significant (chi-square test).

(D) Expression levels of *Sox2*, *Nanog* and *Oct4* pluripotency transcripts between negative sorted (gray) and MERVL$^+$Zscan4c$^+$ (dark blue) cells as determined by RNA-sequencing. Bars represent mean + SD of at least three biological replicates.

(E) Oct4 immunofluorescence staining of cells labeled by the MERVL::tdTomato reporter (middle panel, red). MERVL$^+$ cells are outlined in white dotted lines. Scale bar represents 10 μm.

(F) SUnSET assay showing sites of active translation by a puromycin pulse and detected using a puromycin antibody (second panel, red) in cells labeled with the Zscan4c::EGFP reporter (first panel, green). DNA is visualized with DAPI (gray). Zscan4c$^+$ cells are outlined in white dotted lines. Scale bar represents 10 μm.

(G) Semiquantitative analysis of SUnSET assay. Percentage of cells scored as having high (dark gray) or low (light gray) levels of puromycin immunofluorescence. Subsequently, cells were scored based on either the Zscan4c::EGFP or MERVL::tdTomato as negative cells or MERVL$^+$Zscan4c$^+$ cells.

See also Figure S5.

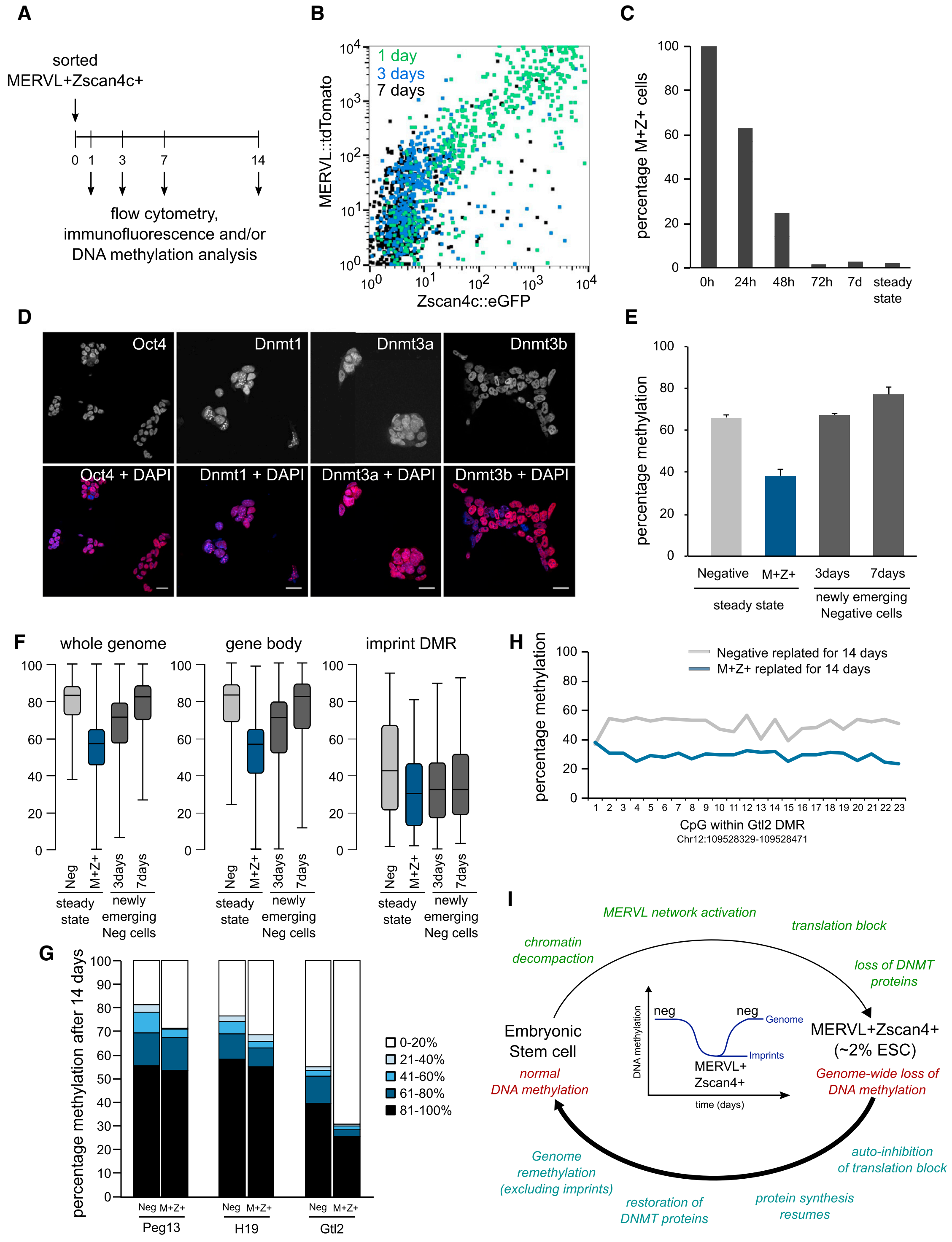

Figure 7. Epigenetic Consequences of Passing through the MERVL$^+$Zscan4$^+$ State

(A) Schematic showing release experiment in which MERVL$^+$Zscan4c$^+$ cells were purified from the steady-state population by flow cytometry sorting of the Zscan4c$^+$ MERVL$^+$ gate, followed by re-culturing. At the indicated time points, cells were re-analyzed by flow cytometry and the Zscan4c/MERVL-negative population collected for immunofluorescence and/or methylation analysis.

(B) Flow cytometry profiles of sorted MERVL$^+$Zscan4c$^+$ (M$^+$Z$^+$) cells following re-culturing for 24 hr (green), 72 hr (blue), or 7 days (black).

(C) Quantification of the proportion of cells remaining in the MERVL$^+$Zscan4$^+$ state (MERVL$^+$ and/or Zscan4c$^+$) at various time points following re-culturing of the sorted cells.

(legend continued on next page)

are to be studied functionally and/or used to generate genetically modified mice.

Our single-cell transcriptome analyses reveal the dynamics of MERVL network activation. By ordering the cells by pseudotime, we were able to identify the earliest transcripts upregulated as cells enter the MERVL$^+$Zscan4$^+$ state, which importantly included MERVL, supporting its role as a driver. Following MERVL activation, the remainder of the transcriptional network is activated in subsequent waves, including a final group of potential repressors that may act to enable the cell to exit the MERVL$^+$Zscan4$^+$ state.

In addition to ESCs, activation of the MERVL-LTR-driven transcriptional network occurs in preimplantation embryos at the time of zygotic genome activation (two-cell stage). This has led to the cells being called "2C-like" and claims of increased potency made (Ishiuchi et al., 2015; Macfarlan et al., 2012). Furthermore, the increased chromatin accessibility in MERVL$^+$Zscan4$^+$ cells is pronounced at MERVL elements, consistent with the open chromatin at active MERVLs in two-cell embryos (Wu et al., 2016). Importantly, we show that the network is activated *in vitro* during the early stages of iPSC reprogramming, suggesting that it may be a general feature of cell identity change, although it remains unknown if this network is upregulated *in vivo* at other developmental stages or species. Interestingly, forced overexpression of Zscan4 increases the efficiency of iPSC reprogramming and increases the expression of members of the MERVL-LTR-driven transcriptional network (Hirata et al., 2012).

Given the dynamic nature of MERVL$^+$Zscan4$^+$ cells, the extent and generality of DNA hypomethylation was striking. The demethylation occurred globally across all genomic features and importantly occurs as a consequence of retroviral network activation and not *vice versa*. Consistently, treatment of ESCs with 5-azacytidine, which induces DNA demethylation, does not lead to activation of MERVL-LTR-driven transcripts (Macfarlan et al., 2011). Our hairpin bisulfite analysis revealed that a reduced activity of the maintenance methyltransferase Dnmt1 and the absence of the *de novo* methyltransferases Dnmt3a and Dnmt3b could explain the observed demethylation. Indeed, there was a dramatic depletion in the Dnmt proteins in MERVL$^+$Zscan4c$^+$ cells, despite similar transcript levels. We confirmed a global reduction of translation in MERVL$^+$Zscan4$^+$ cells (Hung et al., 2013), which is likely the mechanism through which depletion of Dnmt and pluripotency proteins, including Oct4 (Ishiuchi et al., 2015; Macfarlan et al., 2012), occurs, although it is possible that additional pathways, including the altered cell cycle of MERVL$^+$Zscan4$^+$ cells, could also be involved. Interestingly, as the cluster of Eif1a-like transcripts that have been proposed to act in a dominant-negative manner to suppress protein synthesis (Hung et al., 2013) are among the earliest group of transcripts upregulated in MERVL$^+$Zscan4c$^+$ cells (cluster 2 in Figure 1E), transcripts expressed from later clusters may not be translated in MERVL$^+$Zscan4$^+$ cells.

In conclusion, we provide detailed molecular insights into the dynamics of an endogenous retroviral transcriptional network activated in rare mouse ESCs, preimplantation embryos and during iPSC reprogramming. Our studies also demonstrate the downstream effects of activating this network, including global DNA demethylation. While methylation levels are restored once cells exit the MERVL$^+$Zscan4$^+$ state, genomic imprints are not. In summary, our study provides a detailed molecular understanding of the transcriptional dynamics and epigenetic characteristics of MERVL$^+$Zscan4$^+$ cells and potential functional consequences of this dynamic transient ESC state.

EXPERIMENTAL PROCEDURES

Cell Culture

E14 ESCs were cultured in DMEM containing 15% fetal calf serum and 10^3 U/mL leukemia inhibitory factor (LIF); for 2i/LIF experiments, cells were cultured in serum-free N2B27 supplemented with LIF, 1 μM PD0325901, and 3 μM CHIR99021 inhibitors. The Zscan4c::emerald plasmid (90636) was kindly provided by Minoru Ko (Zalzman et al., 2010), and the MERVL::tdTomato reporter (Macfarlan et al., 2012) was a gift from Samuel Pfaff (Addgene #40281). Reporter stable clonal lines were generated by transfection (Fugene6, Promega), drug selection, and subcloning. Flow cytometry analysis was performed using the BD LSR Fortessa and sorts performed on the BD Aria III or BD Influx High-Speed Cell Sorter.

(D) Immunofluorescence staining of sorted MERVL$^+$Zscan4c$^+$ cells that were re-cultured for 14 days. Top row shows Oct4 (first column), Dnmt1 (second column), Dnmt3a (third column), and Dnmt3b (last column). Bottom row depicts merge with DAPI in blue. Scale bar represents 25 μm.

(E) Genome-wide methylation levels determined by PBAT analysis. Steady-state negative-sorted (light gray) and MERVL$^+$Zscan4c$^+$ sorted (M$^+$Z$^+$, dark blue) cells are shown in the first two columns. The last two columns (dark gray) reflect cells that have exited the MERVL$^+$Zscan4c$^+$ state. To identify these, MERVL$^+$Zscan4c$^+$ cells were isolated by flow cytometry and returned to culture. After 3 or 7 days, cells were sorted again. Those lacking MERVL/Zscan4c reporter expression are defined as newly emerging negative cells. Bars represent the mean ± SD of three biological replicates of 100 cells each.

(F) Box plots showing distribution of methylation levels for whole-genome, gene bodies, and imprint DMRs. Steady-state negative-sorted (light gray) and MERVL$^+$Zscan4c$^+$ sorted (M$^+$Z$^+$, dark blue) cells are shown in the first two columns for each feature analyzed. The last two columns (dark gray) reflect cells that have exited the MERVL$^+$Zscan4c$^+$ state identified as in (E) above.

(G) Methylation analysis of Peg13, H19, and Gtl2 imprinted DMRs. Cells were sorted from either negative gates (first of bar pairs) or as MERVL$^+$Zscan4c$^+$ positive (second of bar pairs) and returned to culture for 14 days, and analysis was performed on the resulting bulk population. Imprint DMRs were amplified from bisulfite-treated DNA and sequenced giving >1,000-fold coverage. Reads were binned based on their total amount of methylation (0%–20% white, 21%–40% light blue, 41%–60% medium blue, 61%–80% dark blue, and 81%–100% black).

(H) Average methylation levels for each CpG within Gtl2 DMR for negative (gray) or MERVL$^+$Zscan4c$^+$ (M$^+$Z$^+$, blue) cells that had been initially sorted and then replated for 14 days.

(I) MERVL endogenous retrovirus activation induces genome-wide DNA demethylation. Embryonic stem cells cycle in and out of the MERVL$^+$Zscan4$^+$ state. Entry into the state is initiated by chromatin decompaction, which enables accessibility of transcription factors, resulting in activation of the MERVL-LTR-driven transcriptional network. Subsequently, inhibition of protein synthesis depletes DNMT protein levels in the cell, which results in genome-wide DNA demethylation. Upon exit from the MERVL$^+$Zscan4$^+$ state, the translation block is lifted through auto-inhibition, protein synthesis resumes, and DMNT protein levels return to normal. While genome-wide methylation levels are restored, genomic imprints remain unmethylated, potentially explaining how imprints are lost in long-term ESC cultures.

RNA Isolation, qPCR, and Total RNA-Sequencing

RNA was isolated using QIAGEN RNA-DNA allprep columns or TriReagent (Sigma) and treated with DNaseI (Ambion). cDNA was generated using 0.5–1 μg RNA (Thermo RevertAid) and qRT-PCR performed using the Brilliant III SYBR mix (Agilent Technologies). Relative quantification was performed using the comparative CT method with normalization to CycloB1 levels. Primer sequences available upon request. Opposite strand-specific total RNA libraries (ribozero) were made using 200 ng to 1 μg DNase-treated RNA using the Sanger Institute Illumina bespoke pipeline. ~6–8 × 10^6 paired-end 75-bp reads were generated per sample (at least three biological replicates each) using the Illumina HiSeq 2500 Rapid Run platform.

Whole-Genome Bisulfite Sequencing

Whole-genome bisulfite sequencing libraries were generated using a post-bisulfite adaptor tagging (PBAT) method as previously described (Peat et al., 2014) with ten cycles of amplification. Three biological replicates were generated per condition and libraries sequenced using Illumina HiSeq 2000.

For additional methods, including immunofluorescence, ATAC-seq, and full bioinformatics analysis and statistical methods, see the Supplemental Experimental Procedures.

ACCESSION NUMBERS

The accession numbers for the datasets reported in this paper are GEO: GSE75751, GEO: GSE85776, and ArrayExpress: E-MTAB-5058 (single-cell RNA-seq data).

SUPPLEMENTAL INFORMATION

Supplemental Information includes Supplemental Experimental Procedures, six figures, and four tables and can be found with this article online at http://dx.doi.org/10.1016/j.celrep.2016.08.087.

AUTHOR CONTRIBUTIONS

M.A.E.-M. and W.R. conceived and designed the study. M.A.E.-M. performed experiments, analyzed data, and wrote the paper. V.S. helped design and performed all analysis on single-cell RNA-seq experiments. C. Krueger performed repeat transcriptome analysis and edited the paper. T.M.S. helped design and perform low-cell-number PBAT experiments. P.G. performed hairpin bisulfite experiments. F.K. performed bioinformatics processing. C. Kyriakopoulos performed hairpin bisulfite modelling. R.J.M. prepared single-cell RNA-seq libraries. R.V.B. performed Dnmt1 knockout experiments. I.M. performed iPSC experiments. J.W., S.A.T., and W.R. supervised the study.

ACKNOWLEDGMENTS

The authors thank all members of the W.R. laboratory for helpful discussions. We also thank Ferdinand von Meyenn and Mario Iurlaro for serum-2i transcriptome data, Simon Andrews for bioinformatics support, Simon Walker for imaging support, Nathalie Smerdon for high-throughput sequencing assistance, David Oxley for mass spectrometry, Rachael Walker for flow cytometry assistance, Ruslan Strogantsev for genomic imprinting discussions, Minoru Ko for the Zscan4c::EGFP plasmid, Samuel Pfaff for the MERVL::tdTomato plasmid, and Haruhiko Koseki for Dnmt1$^{fl/fl}$ ESCs. M.A.E.-M. is supported by an EMBO Fellowship (ALTF938-2014) and Marie Sklodowska-Curie Individual Fellowship. P.G. was supported by DFG grant WA1029 within the SFB1027. Research in W.R.'s lab is supported by the BBSRC (grant BB/K010867/1), Wellcome Trust (grant 095645/Z/11/Z), EU EpiGeneSys, and BLUEPRINT.

Received: June 3, 2016
Revised: July 19, 2016
Accepted: August 25, 2016
Published: September 27, 2016

REFERENCES

Akiyama, T., Xin, L., Oda, M., Sharov, A.A., Amano, M., Piao, Y., Cadet, J.S., Dudekula, D.B., Qian, Y., Wang, W., et al. (2015). Transient bursts of Zscan4 expression are accompanied by the rapid derepression of heterochromatin in mouse embryonic stem cells. DNA Res. *22*, 307–318.

Arand, J., Spieler, D., Karius, T., Branco, M.R., Meilinger, D., Meissner, A., Jenuwein, T., Xu, G., Leonhardt, H., Wolf, V., and Walter, J. (2012). In vivo control of CpG and non-CpG DNA methylation by DNA methyltransferases. PLoS Genet. *8*, e1002750.

Barlow, D.P., and Bartolomei, M.S. (2014). Genomic imprinting in mammals. Cold Spring Harb. Perspect. Biol. *6*, a018382.

Bošković, A., Eid, A., Pontabry, J., Ishiuchi, T., Spiegelhalter, C., Raghu Ram, E.V.S., Meshorer, E., and Torres-Padilla, M.-E. (2014). Higher chromatin mobility supports totipotency and precedes pluripotency in vivo. Genes Dev. *28*, 1042–1047.

Buettner, F., Natarajan, K.N., Casale, F.P., Proserpio, V., Scialdone, A., Theis, F.J., Teichmann, S.A., Marioni, J.C., and Stegle, O. (2015). Computational analysis of cell-to-cell heterogeneity in single-cell RNA-sequencing data reveals hidden subpopulations of cells. Nat. Biotechnol. *33*, 155–160.

Dan, J., Yang, J., Liu, Y., Xiao, A., and Liu, L. (2015). Roles for histone acetylation in regulation of telomere elongation and two-cell state in mouse ES cells. J. Cell. Physiol. *230*, 2337–2344.

De Los Angeles, A., Ferrari, F., Xi, R., Fujiwara, Y., Benvenisty, N., Deng, H., Hochedlinger, K., Jaenisch, R., Lee, S., Leitch, H.G., et al. (2015). Hallmarks of pluripotency. Nature *525*, 469–478.

Dean, W., Bowden, L., Aitchison, A., Klose, J., Moore, T., Meneses, J.J., Reik, W., and Feil, R. (1998). Altered imprinted gene methylation and expression in completely ES cell-derived mouse fetuses: association with aberrant phenotypes. Development *125*, 2273–2282.

Deng, T., Kuang, Y., Zhang, D., Wang, L., Sun, R., Xu, G., Wang, Z., and Fei, J. (2007). Disruption of imprinting and aberrant embryo development in completely inbred embryonic stem cell-derived mice. Dev. Growth Differ. *49*, 603–610.

Domcke, S., Bardet, A.F., Adrian Ginno, P., Hartl, D., Burger, L., and Schübeler, D. (2015). Competition between DNA methylation and transcription factors determines binding of NRF1. Nature *528*, 575–579.

Falco, G., Lee, S.-L., Stanghellini, I., Bassey, U.C., Hamatani, T., and Ko, M.S.H. (2007). Zscan4: a novel gene expressed exclusively in late 2-cell embryos and embryonic stem cells. Dev. Biol. *307*, 539–550.

Ficz, G., Hore, T.A., Santos, F., Lee, H.J., Dean, W., Arand, J., Krueger, F., Oxley, D., Paul, Y.-L., Walter, J., et al. (2013). FGF signaling inhibition in ESCs drives rapid genome-wide demethylation to the epigenetic ground state of pluripotency. Cell Stem Cell *13*, 351–359.

Friedli, M., and Trono, D. (2015). The developmental control of transposable elements and the evolution of higher species. Annu. Rev. Cell Dev. Biol. *31*, 429–451.

Friedli, M., Turelli, P., Kapopoulou, A., Rauwel, B., Castro-Díaz, N., Rowe, H.M., Ecco, G., Unzu, C., Planet, E., Lombardo, A., et al. (2014). Loss of transcriptional control over endogenous retroelements during reprogramming to pluripotency. Genome Res. *24*, 1251–1259.

Fujii, S., Nishikawa-Torikai, S., Futatsugi, Y., Toyooka, Y., Yamane, M., Ohtsuka, S., and Niwa, H. (2015). Nr0b1 is a negative regulator of Zscan4c in mouse embryonic stem cells. Sci. Rep. *5*, 9146.

Habibi, E., Brinkman, A.B., Arand, J., Kroeze, L.I., Kerstens, H.H.D., Matarese, F., Lepikhov, K., Gut, M., Brun-Heath, I., Hubner, N.C., et al. (2013). Whole-genome bisulfite sequencing of two distinct interconvertible DNA methylomes of mouse embryonic stem cells. Cell Stem Cell *13*, 360–369.

Hayashi, M., Maehara, K., Harada, A., Semba, Y., Kudo, K., Takahashi, H., Oki, S., Meno, C., Ichiyanagi, K., Akashi, K., and Ohkawa, Y. (2016). Chd5 regulates MuERV-L/MERVL expression in mouse embryonic stem cells via H3K27me3 modification and histone H3.1/H3.2. J. Cell. Biochem. *117*, 780–792.

Hirata, T., Amano, T., Nakatake, Y., Amano, M., Piao, Y., Hoang, H.G., and Ko, M.S.H. (2012). Zscan4 transiently reactivates early embryonic genes during the generation of induced pluripotent stem cells. Sci. Rep. *2*, 208.

Hisada, K., Sánchez, C., Endo, T.A., Endoh, M., Román-Trufero, M., Sharif, J., Koseki, H., and Vidal, M. (2012). RYBP represses endogenous retroviruses and preimplantation- and germ line-specific genes in mouse embryonic stem cells. Mol. Cell. Biol. *32*, 1139–1149.

Humpherys, D., Eggan, K., Akutsu, H., Hochedlinger, K., Rideout, W.M., 3rd, Biniszkiewicz, D., Yanagimachi, R., and Jaenisch, R. (2001). Epigenetic instability in ES cells and cloned mice. Science *293*, 95–97.

Hung, S.S.C., Wong, R.C.B., Sharov, A.A., Nakatake, Y., Yu, H., and Ko, M.S.H. (2013). Repression of global protein synthesis by Eif1a-like genes that are expressed specifically in the two-cell embryos and the transient Zscan4-positive state of embryonic stem cells. DNA Res. *20*, 391–402.

Ishiuchi, T., and Torres-Padilla, M.-E. (2013). Towards an understanding of the regulatory mechanisms of totipotency. Curr. Opin. Genet. Dev. *23*, 512–518.

Ishiuchi, T., Enriquez-Gasca, R., Mizutani, E., Bošković, A., Ziegler-Birling, C., Rodriguez-Terrones, D., Wakayama, T., Vaquerizas, J.M., and Torres-Padilla, M.-E. (2015). Early embryonic-like cells are induced by downregulating replication-dependent chromatin assembly. Nat. Struct. Mol. Biol. *22*, 662–671.

Kigami, D., Minami, N., Takayama, H., and Imai, H. (2003). MuERV-L is one of the earliest transcribed genes in mouse one-cell embryos. Biol. Reprod. *68*, 651–654.

Kolodziejczyk, A.A., Kim, J.K., Tsang, J.C.H., Ilicic, T., Henriksson, J., Natarajan, K.N., Tuck, A.C., Gao, X., Bühler, M., Liu, P., et al. (2015). Single cell RNA-sequencing of pluripotent states unlocks modular transcriptional variation. Cell Stem Cell *17*, 471–485.

Kumar, R.M., Cahan, P., Shalek, A.K., Satija, R., DaleyKeyser, A.J., Li, H., Zhang, J., Pardee, K., Gennert, D., Trombetta, J.J., et al. (2014). Deconstructing transcriptional heterogeneity in pluripotent stem cells. Nature *516*, 56–61.

Lee, H.J., Hore, T.A., and Reik, W. (2014). Reprogramming the methylome: erasing memory and creating diversity. Cell Stem Cell *14*, 710–719.

Leitch, H.G., McEwen, K.R., Turp, A., Encheva, V., Carroll, T., Grabole, N., Mansfield, W., Nashun, B., Knezovich, J.G., Smith, A., et al. (2013). Naive pluripotency is associated with global DNA hypomethylation. Nat. Struct. Mol. Biol. *20*, 311–316.

Macfarlan, T.S., Gifford, W.D., Agarwal, S., Driscoll, S., Lettieri, K., Wang, J., Andrews, S.E., Franco, L., Rosenfeld, M.G., Ren, B., and Pfaff, S.L. (2011). Endogenous retroviruses and neighboring genes are coordinately repressed by LSD1/KDM1A. Genes Dev. *25*, 594–607.

Macfarlan, T.S., Gifford, W.D., Driscoll, S., Lettieri, K., Rowe, H.M., Bonanomi, D., Firth, A., Singer, O., Trono, D., and Pfaff, S.L. (2012). Embryonic stem cell potency fluctuates with endogenous retrovirus activity. Nature *487*, 57–63.

Maksakova, I.A., Thompson, P.J., Goyal, P., Jones, S.J., Singh, P.B., Karimi, M.M., and Lorincz, M.C. (2013). Distinct roles of KAP1, HP1 and G9a/GLP in silencing of the two-cell-specific retrotransposon MERVL in mouse ES cells. Epigenetics Chromatin *6*, 15.

Park, S.-J., Shirahige, K., Ohsugi, M., and Nakai, K. (2015). DBTMEE: a database of transcriptome in mouse early embryos. Nucleic Acids Res. *43*, D771–D776.

Peat, J.R., Dean, W., Clark, S.J., Krueger, F., Smallwood, S.A., Ficz, G., Kim, J.K., Marioni, J.C., Hore, T.A., and Reik, W. (2014). Genome-wide bisulfite sequencing in zygotes identifies demethylation targets and maps the contribution of TET3 oxidation. Cell Rep. *9*, 1990–2000.

Rowe, H.M., Jakobsson, J., Mesnard, D., Rougemont, J., Reynard, S., Aktas, T., Maillard, P.V., Layard-Liesching, H., Verp, S., Marquis, J., et al. (2010). KAP1 controls endogenous retroviruses in embryonic stem cells. Nature *463*, 237–240.

Schlesinger, S., and Goff, S.P. (2015). Retroviral transcriptional regulation and embryonic stem cells: war and peace. Mol. Cell. Biol. *35*, 770–777.

Scialdone, A., Natarajan, K.N., Saraiva, L.R., Proserpio, V., Teichmann, S.A., Stegle, O., Marioni, J.C., and Buettner, F. (2015). Computational assignment of cell-cycle stage from single-cell transcriptome data. Methods *85*, 54–61.

Sharif, J., Endo, T.A., Nakayama, M., Karimi, M.M., Shimada, M., Katsuyama, K., Goyal, P., Brind'Amour, J., Sun, M.A., Sun, Z., et al. (2016). Activation of endogenous retroviruses in Dnmt1(-/-) ESCs involves disruption of SETDB1-mediated repression by NP95 binding to hemimethylated DNA. Cell Stem Cell. *19*, 81–94.

Storm, M.P., Kumpfmueller, B., Bone, H.K., Buchholz, M., Sanchez Ripoll, Y., Chaudhuri, J.B., Niwa, H., Tosh, D., and Welham, M.J. (2014). Zscan4 is regulated by PI3-kinase and DNA-damaging agents and directly interacts with the transcriptional repressors LSD1 and CtBP2 in mouse embryonic stem cells. PLoS ONE *9*, e89821.

Sun, B., Ito, M., Mendjan, S., Ito, Y., Brons, I.G.M., Murrell, A., Vallier, L., Ferguson-Smith, A.C., and Pedersen, R.A. (2012). Status of genomic imprinting in epigenetically distinct pluripotent stem cells. Stem Cells *30*, 161–168.

Thompson, P.J., Dulberg, V., Moon, K.-M., Foster, L.J., Chen, C., Karimi, M.M., and Lorincz, M.C. (2015). hnRNP K coordinates transcriptional silencing by SETDB1 in embryonic stem cells. PLoS Genet. *11*, e1004933.

Torres-Padilla, M.E., and Chambers, I. (2014). Transcription factor heterogeneity in pluripotent stem cells: a stochastic advantage. Development *141*, 2173–2181.

von Meyenn, F., Iurlaro, M., Habibi, E., Liu, N.Q., Salehzadeh-Yazdi, A., Santos, F., Petrini, E., Milagre, I., Yu, M., Xie, Z., et al. (2016). Impairment of DNA methylation maintenance is the main cause of global demethylation in naive embryonic stem cells. Mol. Cell *62*, 848–861.

Walter, M., Teissandier, A., Pérez-Palacios, R., and Bourc'his, D. (2016). An epigenetic switch ensures transposon repression upon dynamic loss of DNA methylation in embryonic stem cells. eLife *5*, e11418.

Wu, J., Huang, B., Chen, H., Yin, Q., Liu, Y., Xiang, Y., Zhang, B., Liu, B., Wang, Q., Xia, W., et al. (2016). The landscape of accessible chromatin in mammalian preimplantation embryos. Nature *534*, 652–657.

Zalzman, M., Falco, G., Sharova, L.V., Nishiyama, A., Thomas, M., Lee, S.-L., Stagg, C.A., Hoang, H.G., Yang, H.-T., Indig, F.E., et al. (2010). Zscan4 regulates telomere elongation and genomic stability in ES cells. Nature *464*, 858–863.

Cell Reports, Volume *17*

Supplemental Information

MERVL/Zscan4 Network Activation Results in Transient Genome-wide DNA Demethylation of mESCs

Mélanie A. Eckersley-Maslin, Valentine Svensson, Christel Krueger, Thomas M. Stubbs, Pascal Giehr, Felix Krueger, Ricardo J. Miragaia, Charalampos Kyriakopoulos, Rebecca V. Berrens, Inês Milagre, Jörn Walter, Sarah A. Teichmann, and Wolf Reik

Supplementary Information

Supplementary Experimental Procedures

Cell Culture medium composition

Serum/LIF: DMEM 4,500 mg/l glucose, 4 mM L-glutamine, 110 mg/l sodium pyruvate, 15% fetal bovine serum, 1 U/ml penicillin, 1 mg/ml streptomycin, 0.1 mM nonessential amino acids, 50 mM b-mercaptoethanol, and 10^3 U/ml LIF.
2i/LIF: 50:50 DMEM/F12:Neurobasal medium (Gibco), 1x N2 supplement (Gibco), 1x B27 supplement (Gibco), L-glutamine, 1 U/ml penicillin, 1 mg/ml streptomycin, 50 mM b-mercaptoethanol, 10^3 U/ml LIF, 1μM PD0325901 inhibitor and 3μM CHIR99021 inhibitor.

Immunofluorescence

Cells were grown on gelatin-coated glass coverslips, fixed in 4% formaldehyde (Polysciences, Inc. Cat #18814) for 20-30 minutes and permeabilised in 0.5% Triton X-100 in PBS for 15-20 minutes at room temperature. Coverslips were blocked in 3% BSA for 1-3 hours at room temperature or overnight at 4 degrees. Primary antibodies were used at the following dilutions: anti-Oct4 (Santa Cruz sc5279) 1:100; anti-Nanog (abcam ab80892) 1:100; anti-Sox2 (abcam ab97959) 1:100; anti-Dnmt1 (abcam ab87654) 1:100; anti-Dnmt3a (Santa Cruz sc20703) 1:50; anti-Dnmt3b (abcam ab13604) 1:500; anti-Dppa4 (Santa Cruz sc74616 1:200). The SUnSET assay was performed as previously described (Schmidt et al., 2009), with a pre-incubation of 1μg/ml puromycin for 10minutes at 37 degrees prior to fixation and detection using anti-puromycin antibody (Millipore MABE343 clone 12D10) at 1:200 dilution. Fluorescently labelled (Alexa488 or Alexa647) secondary antibodies were used at 1:1000 for 30-60 minutes at room temperature, coverslips counterstained with DAPI and mounted in ProLong Gold Antifade mounting medium (Invitrogen P36934). Images were acquired using a Zeiss 780 confocal microscope system with 40x or 63x oil immersion lenses. Image processing was performed using Zeiss ZEN or FUJI software. For semi-quantitative analysis of DNA organisation, nuclei were classified as normal (containing discrete heterochromatic foci) or altered (few large DAPI dense regions), based on DAPI staining. Subsequently, the Zscan4+ or MERVL+ status of the cell was determined.

ATAC-seq analysis

Raw FastQ data were trimmed with Trim Galore to remove Nextera adapters and poor basecall qualities (v0.4.1, default parameters) and mapped to the mouse GRCm38 genome assembly using Bowtie 2 (v2.2.5). Data were quantitated using SeqMonk (www.bioinformatics.babraham.ac.uk/projects/seqmonk/). For promoter analysis, 5kb probes surrounding transcription start site (TSS) were defined and probe trend plots generated. For the bean plots in figure 1F and S1D, probes were generated for each of the features, normalised read counts performed, probe reports generated and RStudio used for plotting (genome: 500bp sliding windows; genic: annotated genes +/- 2kb; inergenic: whole genome – genic regions; promoters: 1kb upstream of TSS; upregulated promoters: promoters of genes differentially expressed as determined by RNA-seq analysis (see below); ERVL, MT2_Mm, MERVL-int, MT2B, MERVL 2A-int, ERVK and LINE L1: appropriate RepeatMasker annotation tracks; CpG islands: SeqMonk annotation track; H3K27ac and H3K4me1 enhancers: peak data from (Creyghton et al., 2010); ESC Super Enhancers: data from (Whyte et al., 2013)). For repeat analysis used in Figure S3D, probes were generated for the filtered repeat track used in RNA sequencing repeat analysis (see below) and normalised read counts (with probe length correction) generated.

RNA sequencing data analysis

Raw FastQ data were trimmed with Trim Galore (v0.4.1, default parameters) and mapped to the mouse GRCm38 genome assembly using TopHat v2.0.12, guided by gene models from Ensembl v70. Data were quantitated at mRNA level using the RNA-seq quantitation pipeline in SeqMonk software (www.bioinformatics.babraham.ac.uk/projects/seqmonk/). Strand specific quantification was performed using mRNA probes and cumulative distributions matched across samples. Differentially expressed genes were determined using DESeq2 (p-value 0.05, with multiple testing correction) and Intensity difference filter (p-value 0.05, with multiple testing correction), with the high-confidence DE genes defined as the intersection between the two statistical tests. The final list of MERVL-driven DE genes was determined by taking the sum of the three DE gene lists (180 genes). Manual filtering removed 8 genes (Fam190a, Tnfrsf22, Gm11052, Cmah, Arsk, Gm16344, Fgf1, Ac165327.2) as their exons overlapped with MERVL elements which did not continue to be successfully spliced to the remainder of the gene, giving a final high confidence list of 172 MERVL-driven differentially expressed genes used in all downstream analyses.
Repeat annotations for the mouse GRCm38 genome build (generated by RepeatMasker) were downloaded from the UCSC website (Oct 2015). The genomic sequences of all instances of different repeat families (LTR ERV1, LTR ERVL, LINE_L1, LINE_L2 and SINE_B2 etc.) were extracted and concatenated into repeat family

pseudo-genomes, whereby individual repeat instances were padded by ‘NNNNN’ to prevent reads from aligning over artificially created repeat boundaries. Read 1 files of RNA-Seq datasets were then aligned against the repeat genomes using Bowtie (v1.0.1; default parameters) and alignments to repeat families were scored. Graphing and statistics was performed using Excel or RStudio.

Repeat analysis

The analysis of repetitive genomic regions in our data was performed by a dual approach: A) RNA-Seq reads were mapped against the mouse genome build GRCm38 using Tophat (v2.1.0 with Bowtie 2, v2.2.5) specifying that if a read mapped more than once, it would be assigned one of the genomic locations. Repetitive sequences were then analysed using RepeatMasker annotation. For the global analysis, repeat masker annotations were filtered to be at least 100bp long, and to have at least 10 reads either in our own data, or in RNA-seq data published in (Akiyama et al., 2015). For the analysis of individual repeat classes, no filtering was applied. B) We also wanted to include sequences that are not present in the mouse genome assembly, namely major and minor satellites and telomeres. For major and minor satellites we used the sequences specified in (Akiyama et al., 2015). For LINE, SINE, ERV1, MaLR, ERVK and ERVL elements, genomic sequences specified by RepeatMasker (http://www.repeatmasker.org) were downloaded from the UCSC website and concatenated into repeat class pseudo-genomes with individual repeat instances padded by ‘NNNNN’ to prevent reads from aligning over artificially created boundaries. Similarly, the telomeric hexamer repeat was concatenated to a total length of 300 bp. Alignments were performed using Bowtie2 (v2.2.5). Data shown in Figure 1G was created using approach A, data shown in Figure 1F was created using approach B.

Single-cell RNA sequencing sorting strategy

We sorted four 96-well plates where each plate had five populations: 56 cells in a MERVL+Zscan4c+ population, 16 cells in a Zscan4c+ only population, 8 cells in a negative population with slight MERVL expression, 14 cells from a completely negative populations, and 2 empty wells for control. To attempt to combat potential batch effects during the library preparation, wells were transferred to new plates mixing rows from different sorting plates in to same library plate.

Single cell RNA-sequencing library preparation by SMART-seq v2

Single-cells were sorted in 2uL of Lysis Buffer (1:20 solution of RNase Inhibitor (Clontech, cat. no. 2313A or Invitrogen RNAse OUT) in 0.2% v/v Triton X-100 (Sigma-Aldrich, cat. no. T9284) in 96 well plates, spun down and immediately frozen at -80 degrees. Oligo-dT primer, dNTPs (ThermoFisher, cat. no. 10319879) and ERCC RNA Spike-In Mix (1:25,000,000 final dilution, Ambion, cat. no. 4456740) were then added, and Reverse Transcription and PCR were performed as in (Picelli et al., 2014). The cDNA libraries for sequencing were prepared using Nextera XT DNA Sample Preparation Kit (Illumina, cat. no. FC-131-1096), according to the protocol supplied by Fluidigm (PN 100-5950 B1). Libraries from 96 single cells were pooled and purified using AMPure XP beads (Beckman Coulter). Pooled samples were sequenced on an Illumina HiSeq 2500 instrument, using paired-end 100-bp reads.

Single cell RNA-sequencing data analysis and quality control

The gene expressions for every sample were quantified using Salmon version 0.5.0 (Patro et al., 2015) with library type parameter IU, with a transcriptome index built from the Ensembl 78 cDNA annotation (GRCm38 patch 3), together with ERCC transcript sequences. The index also contained 313 sequences for Mus musculus specific repeats from RepBase (Jurka et al., 2005) for assessing potentially transcribed repeats (such as MERVL). By comparing largely technical features with the 8 empty, control wells, in the plates we filtered out 65 of the 376 samples based on ERCC spike in ratio (more than 60%) and number of sequenced read pairs (less than 100,000). The thresholds were consistent with other technical features such as mapping rate, mitochondrial content and number of detected genes. This left 319 samples that we considered as healthy single cells, and used for further analysis.

From the Transcripts per million (TPM) of any sample, we removed the spike-in expression and rescaled the values of the endogenous genes to sum to a million. This *endogenous TPM* represents the relative abundance of a gene within a cell. For differential expression testing we used linear modelling and the likelihood ratio test for significance analysis, where we controlled for the number of observed genes in a sample.

Pseudotime ordering of scRNA-seq data

To investigate the dynamics of the MERVL network, we created a trajectory (or “pseudotime”) over the expression of Zscan4 (summed TPM for Zscan4b-f) and MERVL for the two positive conditions, using a Bayesian Gaussian Process Latent Variable Model (Titsias and Lawrence, 2010). Given the transcriptome data, it is not possible to distinguish individual genomic copies of MERVL and as such the total MERVL expression was analysed. To identify genes with dynamic expression over this trajectory, we fitted two Gaussian Processes

for every gene; one with a Bias kernel (which assumes all expression changes are due to noise), and one with a Squared Exponential + Bias kernel (which can also handle dynamic changes in addition to noise). We ranked genes based on the ratio of the optimized likelihoods for the models. We considered 297 genes significantly dynamic based on this measure. These dynamic genes were then clustered using the Mixtures of Hierarchical Gaussian Processes model to groups of genes with a similar common expression pattern over the trajectory (Hensman et al., 2015).

Cell cycle analysis

To assess what states of cell cycle where represented in the different conditions of scRNA-Seq data we used the Pairs method in the Cyclone tool (Scialdone et al., 2015). We ran the method on the TPM values of the data.

Bisulfite sequencing analysis

Raw sequence reads were trimmed to remove both poor quality calls and adapters using Trim Galore (v0.4.1, www.bioinformatics.babraham.ac.uk/projects/trim_galore/, Cutadapt version 1.8.1, parameters: --paired). Trimmed reads were first aligned to the mouse genome in paired-end mode to be able to use overlapping parts of the reads only once while writing out unmapped singleton reads; in a second step remaining singleton reads were aligned in single-end mode. Alignments were carried out with Bismark v0.14.4 (Krueger and Andrews, 2011) with the following set of parameters: a) paired-end mode: --pbat; b) single-end mode for Read 1: --pbat; c) single-end mode for Read 2: defaults.
Reads were then deduplicated with deduplicate_bismark selecting a random alignment for position that were covered more than once. CpG methylation calls were extracted from the deduplicated mapping output ignoring the first 6bp of each read to reduce the methylation bias typically observed in PBAT libraries using the Bismark methylation extractor (v0.14.4) with the following parameters: a) paired-end mode: --ignore 6 --ignore_r2 6; b) single-end mode: --ignore 6.
Data were quantitated using SeqMonk (www.bioinformatics.babraham.ac.uk/projects/seqmonk/). Probes were defined to contain 50 CpGs each with a minimum read count of 4 and percentage methylation determined on the pooled replicate data. For analysis of specific genome features these were defined as follows: Gene bodies (probes overlapping genes), Promoters (probes overlapping 1000bp upstream of genes), CGI promoters (promoters containing or within 250bp of a CGI), non-CGI promoters (all other promoters), LMRs (Stadler et al., 2011), H3K27ac and H3K4me1 Enhancers (Creyghton et al., 2010), Super-enhancers (Whyte et al., 2013). For repetitive elements, Bismark (v0.14.4, using Bowtie 2, default parameters) was used to map all reads from each data set against consensus sequences constructed from Repbase (Jurka et al., 2005). The methylation level was expressed as the mean of individual CG sites. Graphing and statistics was performed using Excel or RStudio.

Methylation quantification by PBAT

For global methylation level quantification, whole genome bisulfite libraries were generated using a post-bisulfite adapter tagging (PBAT) method using 10 cycles of amplification. Libraries were sequenced at low coverage generating ~20-30x10^5 aligned reads per sample. Raw sequence reads were processed as above. 50kb probes (minimum 1 read, minimum 1 observation) were defined and mean methylation level determined for each sample.

Mass Spectrometry

Genomic DNA quantified using picogreen assay (Invitrogen) was digested using DNA Degradase plus (Zymo Research) for 90 minutes at 37 degrees and analysed by liquid chromatography-tandem mass spectrometry on a LTQ Orbitrap Velos mass spectrometer (Thermo Scientific) fitted with a nanoelectrospray ion-source (Proxeon, Odense, Denmark). Mass spectral data for cytosine, 5-methylcytosine and 5-hydroxymethylcytosine were acquired as previously described (Ficz et al., 2013).

Hairpin Bisulfite sequencing

Hairpin bisulfite sequencing allows the analysis of both DNA strands of one individual chromosome giving detailed information about the methylation patterns of cells. Genomic DNA is cut using 10U of the restriction enzyme Eco471 for major satellites (mSat; GSAT_MM) and murine endogenous retrovirus-like (MERVL) and 10U of BsaWI for a class of Long Interspersed Nuclear Elements (L1mdT). After heat inactivation of the restriction enzymes 200U T4 DNA Ligase, 10mM ATP and 100µM hairpin oligo nucleotide are added directly to the reaction and incubated for overnight at 16°C. The ligation covalently links both DNA strands with each other. In the following step the reaction is subjected to a bisulfite treatment which was performed using the EZ Methylation-GoldTM Kit from Zymo Research. The treated DNA was amplified by PCR to create amplicons for each repetitive element. Sequencing was performed on a MiSeq Illumina platform with 2x300bp. The Analysis was carried out with the BiQAnalyzer HT and python script. To calculate the efficiencies of de novo

and maintenance methylation we applied an extension of the Hidden Markov Model described in (Arand et al., 2012), allowing de-novo methylation to happen in both hemi and unmethylated CpG sites with the same probability. Primer sequences used are as follows:

MERVL-HP
(CGCCCGAGACAAGGTGATTCTAGTTATTATAATGGACAGCGTAGACAAAAGAATGTTTATAATAA
CATACCCAGTAATGGTCAGCACAGGAGAGGTGAAATTTATAATGGCATGACTCGGTTGgwtgggRttat
dddddddatgggRttgwTTCAACCGAGTCATGCCATTATAAATTTCACCTCTCCTGTGCTGACCATTACTG
GGTATGTTATTATAAACATTCTTTTGTCTACGCTGTCCATTATAATAACTAGAATCACCTTGTCTCG
GGCG);
L1HP
(CCCGGGACCAAGATGGCGACCGCTGCTGCTGTGGCTTAGGCCGCCTCCCCAGCCGGGTGGGCACC
TGTCCTttGGaGGGRttATNNNNNNNNATGGGRtttCCGGAGGACAGGTGCCCACCCGGCTGGGGAGGC
GGCCTAAGCCACAGCAGCAGCGGTCGCCATCTTGGTCCCGGG);
mSat-HP
(GgaaaatttagaaatgtttaatgtaggaCGtggaatatggcaagaaaactgaaaatcatgggaaatgagaaacatccacttgtCGacttgaaaaatgaCGaa
atcactaaaaaaCGtgaaaaatgagaaatgcacactgaaggNTgggRTTatNNNNNNNNatgggRTTgNccttcagtgtgcatttctcatttttca
CGttttttagtgatttCGtcatttttcaagtCGacaagtggatgtttctcattttttatgatttttagtttttttgtt).

Statistical Methods

Statistics were performed using Seqmonk (www.bioinformatics.babraham.ac.uk/projects/seqmonk/), Excel, Graphpad prism or RStudio. For mass spectrometry, a paired t-test was used to determine p-value between measurements made from matched MERVL+Zscan4+ and negative sorted cells on different days. For CpG methylation levels determined by PBAT, a homoscedastic two-tailed t-test was used to determine significance between at least 3 biological replicates for each sample. For statistical analysis of transcription of individual genes, expression levels were quantified in the RNA-sequencing data as above and a homoscedastic two-tailed t-test performed between at least 3 biological replicates per sample. Semi-quantitative immunofluorescence measurements were performed using ImageJ in a blind manner and Chi-square test performed in Excel to determine significance.

Supplemental References

Akiyama, T., Xin, L., Oda, M., Sharov, A.A., Amano, M., Piao, Y., Cadet, J.S., Dudekula, D.B., Qian, Y., Wang, W., Ko, S.B.H., Ko, M.S.H., 2015. Transient bursts of Zscan4 expression are accompanied by the rapid derepression of heterochromatin in mouse embryonic stem cells. DNA Res. dsv013. doi:10.1093/dnares/dsv013

Arand, J., Spieler, D., Karius, T., Branco, M.R., Meilinger, D., Meissner, A., Jenuwein, T., Xu, G., Leonhardt, H., Wolf, V., Walter, J., 2012. In vivo control of CpG and non-CpG DNA methylation by DNA methyltransferases. PLoS Genet. 8, e1002750. doi:10.1371/journal.pgen.1002750

Buettner, F., Natarajan, K.N., Casale, F.P., Proserpio, V., Scialdone, A., Theis, F.J., Teichmann, S.A., Marioni, J.C., Stegle, O., 2015. Computational analysis of cell-to-cell heterogeneity in single-cell RNA-sequencing data reveals hidden subpopulations of cells. Nat. Biotechnol. 33, 155–160. doi:10.1038/nbt.3102

Creyghton, M.P., Cheng, A.W., Welstead, G.G., Kooistra, T., Carey, B.W., Steine, E.J., Hanna, J., Lodato, M.A., Frampton, G.M., Sharp, P.A., Boyer, L.A., Young, R.A., Jaenisch, R., 2010. Histone H3K27ac separates active from poised enhancers and predicts developmental state. Proc. Natl. Acad. Sci. U.S.A. 107, 21931–21936. doi:10.1073/pnas.1016071107

Deng, Q., Ramsköld, D., Reinius, B., Sandberg, R., 2014. Single-cell RNA-seq reveals dynamic, random monoallelic gene expression in mammalian cells. Science 343, 193–196. doi:10.1126/science.1245316

Domcke, S., Bardet, A.F., Adrian Ginno, P., Hartl, D., Burger, L., Schübeler, D., 2015. Competition between DNA methylation and transcription factors determines binding of NRF1. Nature 528, 575–579. doi:10.1038/nature16462

Ficz, G., Hore, T.A., Santos, F., Lee, H.J., Dean, W., Arand, J., Krueger, F., Oxley, D., Paul, Y.-L., Walter, J., Cook, S.J., Andrews, S., Branco, M.R., Reik, W., 2013. FGF signaling inhibition in ESCs drives rapid genome-wide demethylation to the epigenetic ground state of pluripotency. Cell Stem Cell 13, 351–359. doi:10.1016/j.stem.2013.06.004

Hensman, J., Rattray, M., Lawrence, N.D., 2015. Fast Nonparametric Clustering of Structured Time-Series. IEEE Trans Pattern Anal Mach Intell 37, 383–393. doi:10.1109/TPAMI.2014.2318711

Jurka, J., Kapitonov, V.V., Pavlicek, A., Klonowski, P., Kohany, O., Walichiewicz, J., 2005. Repbase Update, a database of eukaryotic repetitive elements. Cytogenet. Genome Res. 110, 462–467. doi:10.1159/000084979

Kolodziejczyk, A.A., Kim, J.K., Tsang, J.C.H., Ilicic, T., Henriksson, J., Natarajan, K.N., Tuck, A.C., Gao, X., Bühler, M., Liu, P., Marioni, J.C., Teichmann, S.A., 2015. Single Cell RNA-Sequencing of Pluripotent States Unlocks Modular Transcriptional Variation. Cell Stem Cell 17, 471–485. doi:10.1016/j.stem.2015.09.011

Krueger, F., Andrews, S.R., 2011. Bismark: a flexible aligner and methylation caller for Bisulfite-Seq applications. Bioinformatics 27, 1571–1572. doi:10.1093/bioinformatics/btr167

Kumar, R.M., Cahan, P., Shalek, A.K., Satija, R., DaleyKeyser, A.J., Li, H., Zhang, J., Pardee, K., Gennert, D., Trombetta, J.J., Ferrante, T.C., Regev, A., Daley, G.Q., Collins, J.J., 2014. Deconstructing transcriptional heterogeneity in pluripotent stem cells. Nature 516, 56–61. doi:10.1038/nature13920

Patro, R., Duggal, G., Kingsford, C., 2015. Accurate, fast, and model-aware transcript expression quantification with Salmon, bioRxiv. doi:10.1101/021592

Picelli, S., Faridani, O.R., Björklund, A.K., Winberg, G., Sagasser, S., Sandberg, R., 2014. Full-length RNA-seq from single cells using Smart-seq2. Nat Protoc 9, 171–181. doi:10.1038/nprot.2014.006

Schmidt, E.K., Clavarino, G., Ceppi, M., Pierre, P., 2009. SUnSET, a nonradioactive method to monitor protein synthesis. Nat Meth 6, 275–277. doi:10.1038/nmeth.1314

Scialdone, A., Natarajan, K.N., Saraiva, L.R., Proserpio, V., Teichmann, S.A., Stegle, O., Marioni, J.C., Buettner, F., 2015. Computational assignment of cell-cycle stage from single-cell transcriptome data. Methods 85, 54–61. doi:10.1016/j.ymeth.2015.06.021

Stadler, M.B., Murr, R., Burger, L., Ivanek, R., Lienert, F., Schöler, A., van Nimwegen, E., Wirbelauer, C., Oakeley, E.J., Gaidatzis, D., Tiwari, V.K., Schübeler, D., 2011. DNA-binding factors shape the mouse methylome at distal regulatory regions. Nature 480, 490–495. doi:10.1038/nature10716

Titsias, M.K., Lawrence, N.D., 2010. Bayesian Gaussian Process Latent Variable Model. Proceedings of the th International Conference on Artificial Intelligence and Statistics AISTATS 1–8.

Wang, L., Zhang, J., Duan, J., Gao, X., Zhu, W., Lu, X., Yang, L., Zhang, J., Li, G., Ci, W., Li, W., Zhou, Q., Aluru, N., Tang, F., He, C., Huang, X., Liu, J., 2014. Programming and inheritance of parental DNA methylomes in mammals. Cell 157, 979–991. doi:10.1016/j.cell.2014.04.017

Whyte, W.A., Orlando, D.A., Hnisz, D., Abraham, B.J., Lin, C.Y., Kagey, M.H., Rahl, P.B., Lee, T.I., Young, R.A., 2013. Master transcription factors and mediator establish super-enhancers at key cell identity genes. Cell 153, 307–319. doi:10.1016/j.cell.2013.03.035

Xue, Z., Huang, K., Cai, C., Cai, L., Jiang, C.-Y., Feng, Y., Liu, Z., Zeng, Q., Cheng, L., Sun, Y.E., Liu, J.-Y.,

Horvath, S., Fan, G., 2013. Genetic programs in human and mouse early embryos revealed by single-cell RNA sequencing. Nature 500, 593–597. doi:10.1038/nature12364

Yue, F., Cheng, Y., Breschi, A., Vierstra, J., Wu, W., Ryba, T., Sandstrom, R., Ma, Z., Davis, C., Pope, B.D., Shen, Y., Pervouchine, D.D., Djebali, S., Thurman, R.E., Kaul, R., Rynes, E., Kirilusha, A., Marinov, G.K., Williams, B.A., Trout, D., Amrhein, H., Fisher-Aylor, K., Antoshechkin, I., DeSalvo, G., See, L.-H., Fastuca, M., Drenkow, J., Zaleski, C., Dobin, A., Prieto, P., Lagarde, J., Bussotti, G., Tanzer, A., Denas, O., Li, K., Bender, M.A., Zhang, M., Byron, R., Groudine, M.T., McCleary, D., Pham, L., Ye, Z., Kuan, S., Edsall, L., Wu, Y.-C., Rasmussen, M.D., Bansal, M.S., Kellis, M., Keller, C.A., Morrissey, C.S., Mishra, T., Jain, D., Dogan, N., Harris, R.S., Cayting, P., Kawli, T., Boyle, A.P., Euskirchen, G., Kundaje, A., Lin, S., Lin, Y., Jansen, C., Malladi, V.S., Cline, M.S., Erickson, D.T., Kirkup, V.M., Learned, K., Sloan, C.A., Rosenbloom, K.R., Lacerda de Sousa, B., Beal, K., Pignatelli, M., Flicek, P., Lian, J., Kahveci, T., Lee, D., Kent, W.J., Ramalho-Santos, M., Herrero, J., Notredame, C., Johnson, A., Vong, S., Lee, K., Bates, D., Neri, F., Diegel, M., Canfield, T., Sabo, P.J., Wilken, M.S., Reh, T.A., Giste, E., Shafer, A., Kutyavin, T., Haugen, E., Dunn, D., Reynolds, A.P., Neph, S., Humbert, R., Hansen, R.S., De Bruijn, M., Selleri, L., Rudensky, A., Josefowicz, S., Samstein, R., Eichler, E.E., Orkin, S.H., Levasseur, D., Papayannopoulou, T., Chang, K.-H., Skoultchi, A., Gosh, S., Disteche, C., Treuting, P., Wang, Y., Weiss, M.J., Blobel, G.A., Cao, X., Zhong, S., Wang, T., Good, P.J., Lowdon, R.F., Adams, L.B., Zhou, X.-Q., Pazin, M.J., Feingold, E.A., Wold, B., Taylor, J., Mortazavi, A., Weissman, S.M., Stamatoyannopoulos, J.A., Snyder, M.P., Guigó, R., Gingeras, T.R., Gilbert, D.M., Hardison, R.C., Beer, M.A., Ren, B., Mouse ENCODE Consortium, 2014. A comparative encyclopedia of DNA elements in the mouse genome. Nature 515, 355–364. doi:10.1038/nature13992

Supplemental Figure Legends

Supplemental Figure 1 (related to Figure 1)

(A) Scatterplot showing normalised, log_2 transformed read counts mapping to specific genomic locations of repetitive sequence. Individual repeat classes are highlighted: MERVL (MT2_Mm + MERVL-int, red), LINE L1 (blue) and IAPEZ (IAPEZ-int, black).

(B) Semi-quantitative analysis of DNA organisation. Nuclei were classified according to their DAPI signal as normal (light grey) or altered (dark grey), and expression of the Zscan4 and MERVL reporters. Differences are statistically significant (chi-squared test: Zscan4 χ^2 6.34 x 10^{-113}; MERVL χ^2 5.22 x 10^{-30}).

(C) Chromatin accessibility of different genomic features as determined by ATAC-seq analysis in negative sorted (grey) and MERVL+Zscan4c+ (dark blue) cells. Bars represent mean levels of accessibility.

(D) Log_{10} TPM (transcripts per million) values of MERVL (x-axis) and Zscan4 cluster (y-axis) of single cells from published unsorted mES single cell datasets (Buettner et al., 2015; Kolodziejczyk et al., 2015; Kumar et al., 2014). Only the 204 cells expressing the Zscan4 cluster are shown.

(E) Smoothened heatmap showing expression levels of 172 differentially expressed genes identified by total RNA-sequencing. Each column represents a single-cell (total n=204) that expresses the Zscan4 cluster from published unsorted mES single cell datasets (Buettner et al., 2015; Kolodziejczyk et al., 2015; Kumar et al., 2014). Cells are ordered based on MERVL expression. Median Spearman rank correlation was 0.3 between MERVL and differentially expressed genes, and 0.0 between MERVL and all genes.

(F) Venn diagram showing overlap between differentially expressed genes identified by total RNA-sequencing and dynamic genes over pseudotime (see supplemental experimental procedures for details).

Supplemental Figure 2 (related to Figure 2)

(A-C) Heat map showing relative expression levels of MERVL-LTR driven transcriptional network in early embryos (A,B) and somatic tissues (C). BAT = brown adipose tissue, MEF = mouse embryonic fibroblasts (MEF). Scale bar depicts log relative expression from low (blue) to high (red). Data from (Deng et al., 2014) (A), (Xue et al., 2013) (B) and (Yue et al., 2014) (C).

Supplemental Figure 3 (related to Figure 3)

(A) Consistency of methylation pattern within individual reads containing at least 3 CpGs. Reads were classified as completely methylated (dark grey, p-value 2.3 x10^{-5}), mixed methylation (medium grey, p-value 2.1x10^{-4}) or completely unmethylated (light grey, p-value 6.8x10^{-4}). Error bars represent standard deviation of three biological replicates. *** all three categories of comparisons between negative and MERVL+Zscan4c+ cells are statistically significant (homoscedastic two-tailed t-test).

(B) Bean plots showing distribution of methylation levels for different genome features between negative sorted (grey) and MERVL+Zscan4c+ (blue) cells. Lines represent mean values.

(C) Methylation levels across the Igf2r imprint DMR (chr17:12731191-12752684). Top track shows gene structure of Igf2r (not all gene is shown). Methylation levels for oocyte (red) and sperm (light blue) allow identification of DMR (highlighted in yellow). Oocyte and sperm data from (Wang et al., 2014). Negative ESC (grey) and MERVL+Zscan4c+ ESC (M+Z+, dark blue) tracks show methylation levels across the region (20 CpGs with min coverage of 4 per probe). Bottom track shows overlay between Negative and MERVL+Zscan4c+ datasets.

Supplemental Figure 4 (related to Figure 4)

(A) Flow cytometry analysis showing percentage of MERVL::tdTomato+ and/or Zscan4c::eGFP+ cells in serum and 2i/LIF culture conditions. Differences are statistically significant (2-tailed equal variance t-test, n=4-6).

(B) Quantitative real-time RT-PCR of six MERVL-LTR driven genes in cells cultured in serum or 2i/LIF conditions. Bars represent average of 3 biological replicates +/- standard deviation. Expression levels in serum conditions are set to 1.

(C) Scatterplot showing normalised log_2 transformed read counts for all genes (grey) and MERVL-LTR promoted genes (blue) between wild-type (x-axis) and DNMT TKO (y-axis) ESCs. Data reanalysed from (Domcke et al., 2015).

(D) Flow cytometry plots showing MERVL::tdTomato (y-axis) versus Zscan4c::eGFP (x-axis) reporter expression in steady state conditions (left plot) and of cells sorted from the negative gate (red) and placed back in culture for 6 hours (second plot from left), 24 hours (second plot from right) or 48 hours (right plot). 10,000 cells are shown in each plot. Gates used for subsequent sorting of newly arising MERVL+Zscan4+ cells for methylation analysis are shown in green. The 6 hour time point used a larger gate due to the very small number of newly arising MERVL+Zscan4+ cells.

Supplemental Figure 5 (related to Figure 5)
(A) Immunofluorescence staining of Nanog (top) and Sox2 (bottom) proteins in cells labelled using MERVL::tdTomato reporter (left panel, red). Images represent single confocal images. DNA is stained with DAPI (blue). Scale bar represents 10µm.
(B) Chromosome view of Eif1a-like cluster showing expression levels in negative-sorted (top row) and MERVL+Zscan4c+ (bottom row) cells. Position of genes are denoted (red = sense, blue = antisense), along with opposing strand specific RNA-sequencing reads (sense transcription shows blue, antisense transcription shows red). Bars represent average expression levels of at least 3 biological replicates + standard deviation. Upregulated genes, corresponding to Eif1a-lie genes, are highlighted in red.
(C) Heat map showing expression of genes within cluster on Chromosome 12 (87473449-88356013) between negative-sorted and MERVL+Zscan4c+ cells.

Supplemental Figure 6 (related to Figure 6)
(A) Computation modelling (dashed lines) of hairpin bisulfite data (solid lines) for all biological replicate pairs. Three separate repeat regions were analysed: LINE L1 (top), MERVL (middle) and Major Satellites (bottom) repeats. In each graph, individual reads are classified as fully methylated (orange), hemi-methylated on the upper strand (dark green), hemi-methylated on the lower strand (light green) or unmethylated (blue) reads.

Supplemental Tables

Supplemental Table 1. Expression levels of differentially expressed genes, related to Figure 1.
List of differentially expressed genes between Negative (MERVL-Zscan4-), Zscan4+ only (MERVL-Zscan4+) and MERVL+Zscan4+ sorted cells, along with their chromosome coordinates, ENSEMBL gene ID, Description and average $\log_2$ expression value for the replicates across the three conditions.

Supplemental Table 2. Expression levels of all assessed genes, related to Figure 1.
List of all genes, chromosome coordinates, ENSEMBL gene ID, Description and average $\log_2$ expression value across the Negative (MERVL-Zscan4-), Zscan4+ only (MERVL-Zscan4+) and MERVL+Zscan4+ sorted cells.

Supplemental Table 3. List of genes dynamic over pseudotime, related to Figure 1.
List of transcripts contained within the 5 clusters that are dynamic over pseudotime as determined by single-cell RNA sequencing analysis (see Supplemental Experimental Procedures).

Supplemental Table 4. Expression levels of differentially expressed genes in other datasets, related to Figure 2.
Expression levels for the 172 MERVL-LTR driven genes in the different datasets analysed. (A) Park et al. (B) Deng et al. (C) Xue et al. (D) Milagre et al. (E) Encode. Note that due to differences in library preparation and data analysis it is not appropriate to compare values across datasets.

Supplementary Figure 1

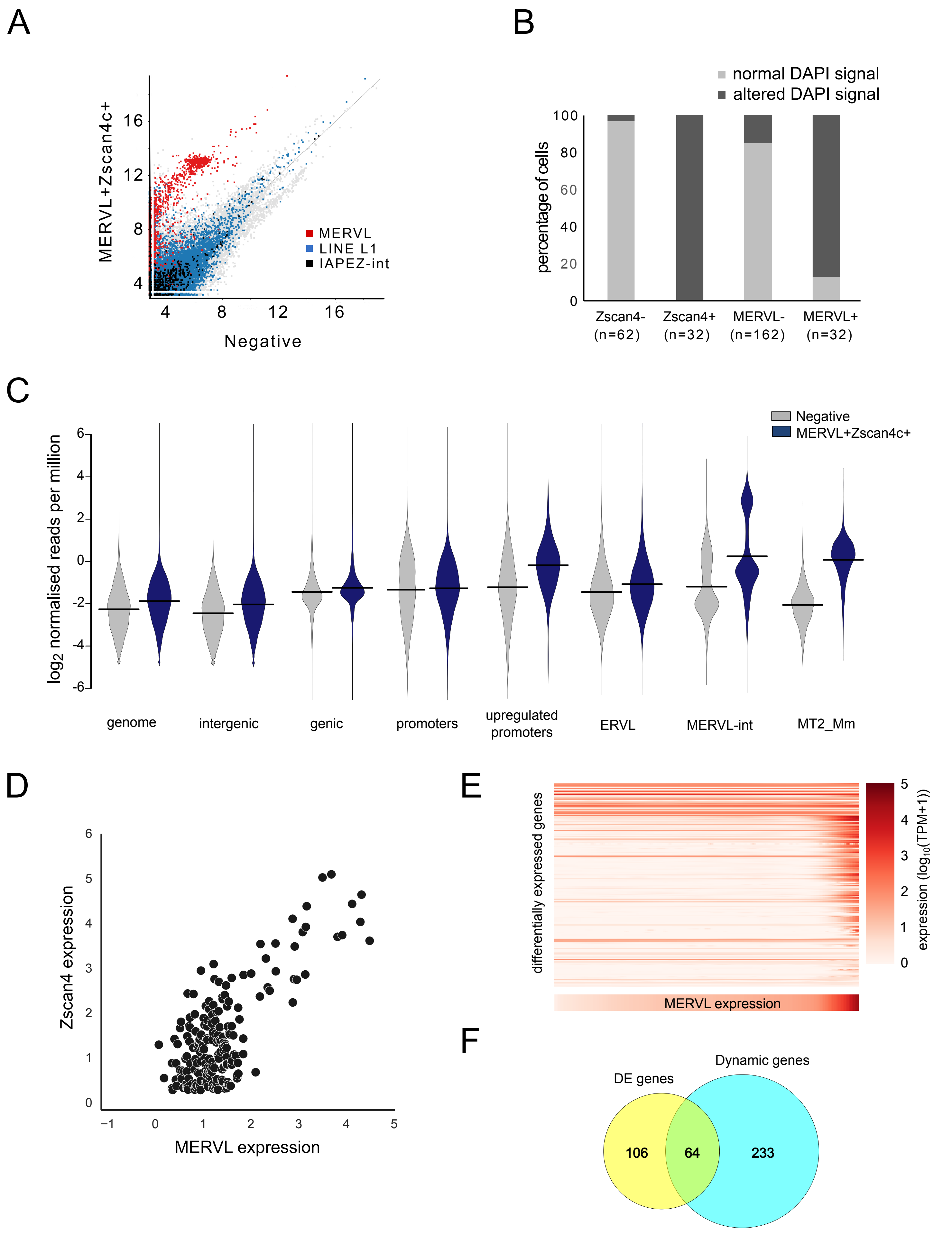

Supplemental Figure 2

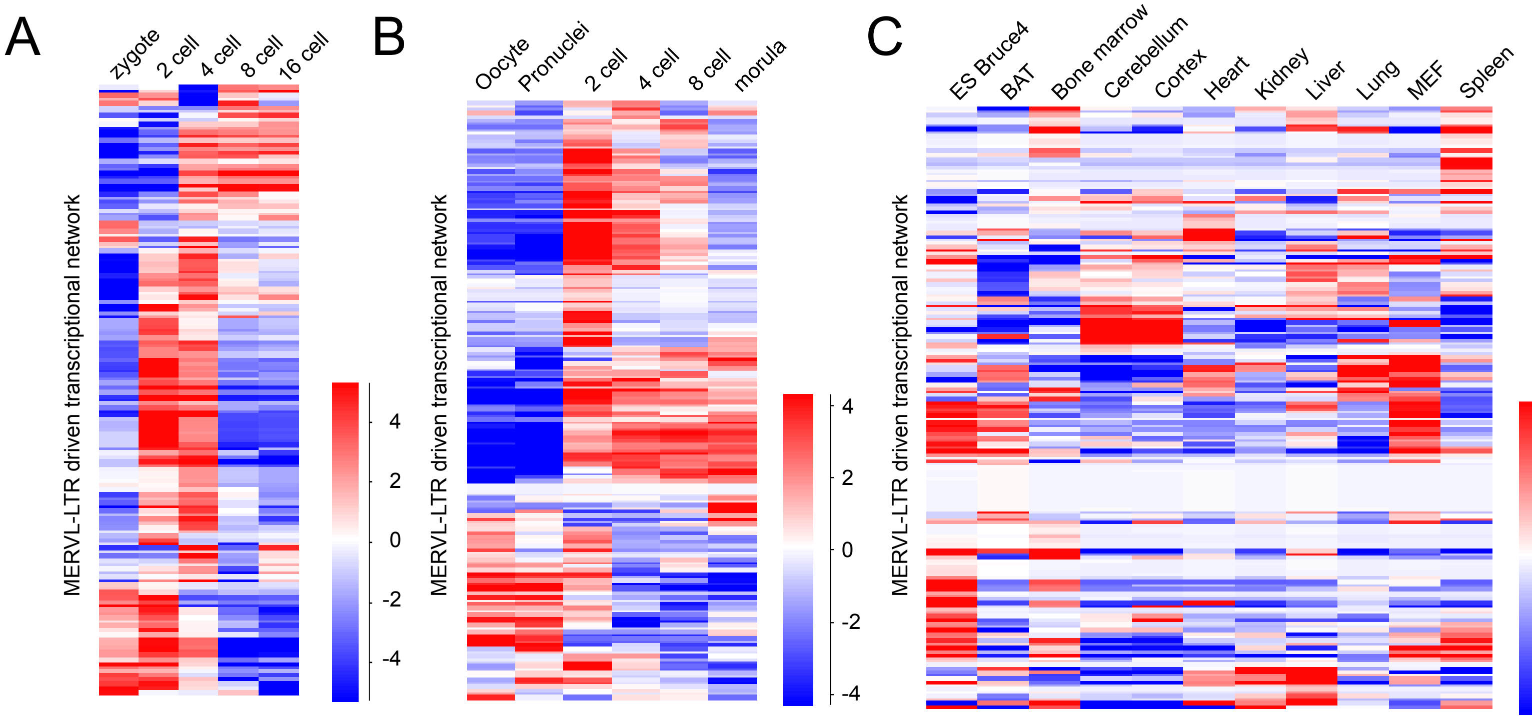

Supplemental Figure 3

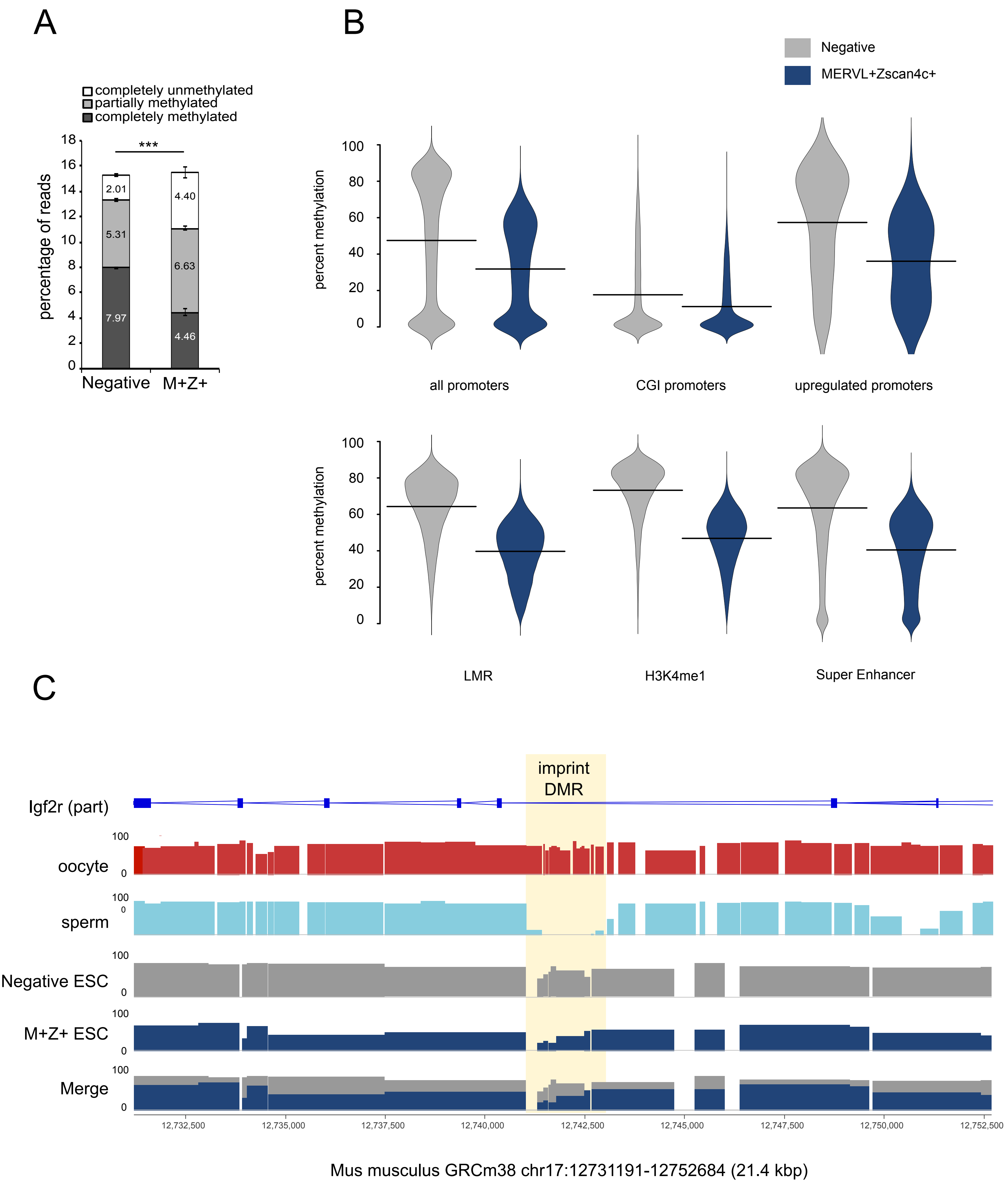

Supplemental Figure 4

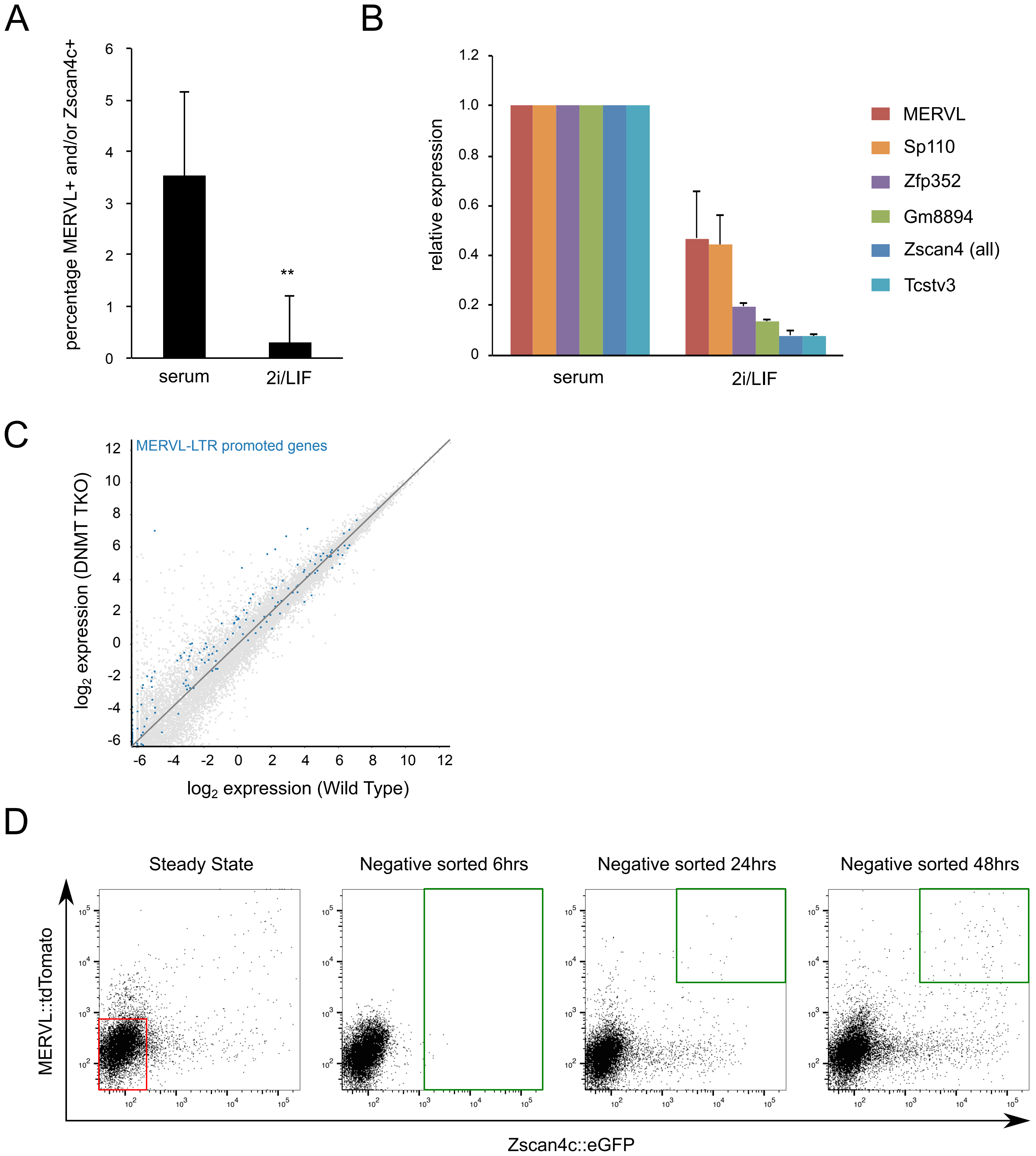

Supplemental Figure 5

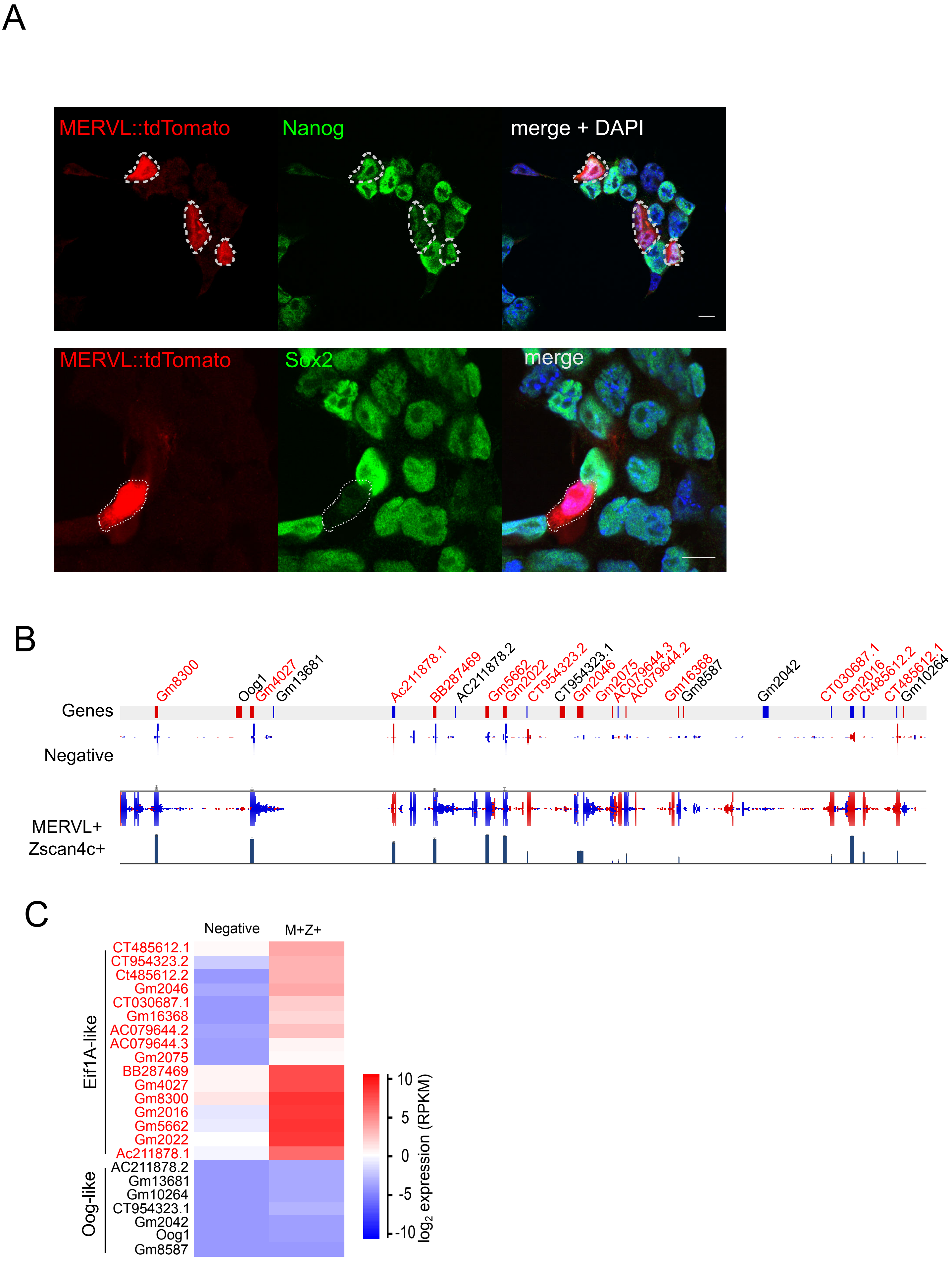

Supplemental Figure 6

A

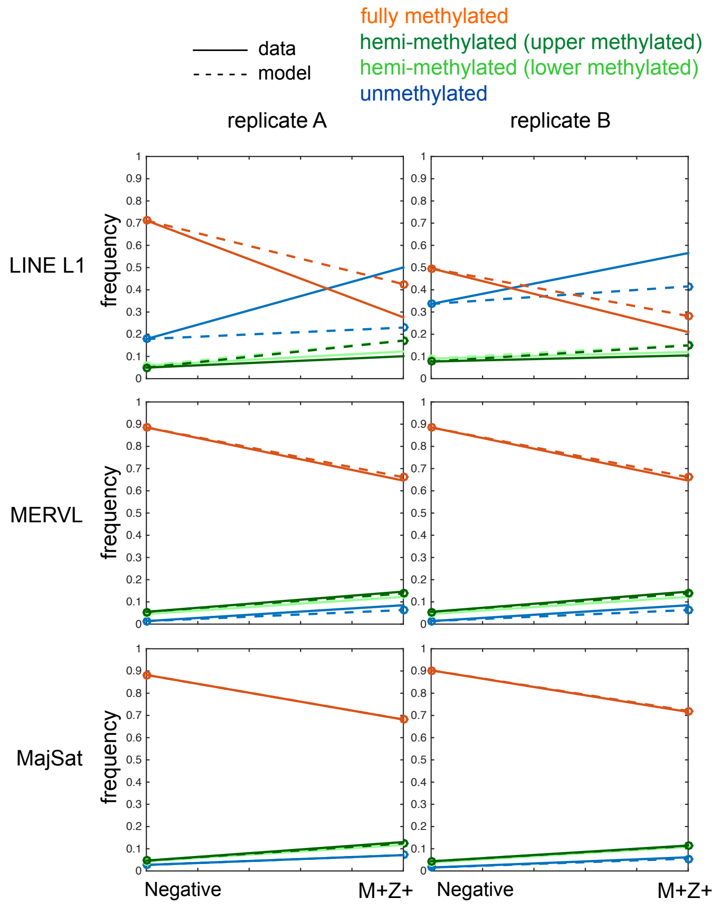

Supplement: Document S2. Article plus Supplemental Information [file mmc6.pdf]
